# Deciphering the impact of *PROM1* alternative splicing on human photoreceptor development and maturation

**DOI:** 10.1038/s41419-024-07105-7

**Published:** 2024-10-01

**Authors:** Marina Moya-Molina, Birthe Dorgau, Emily Flood, Stef J. F. Letteboer, Esben Lorentzen, Jonathan Coxhead, Graham Smith, Ronald Roepman, Sushma Nagaraja Grellscheid, Lyle Armstrong, Majlinda Lako

**Affiliations:** 1 Biosciences Institute, Newcastle University, Newcastle upon Tyne, UK; 2 Newcells Biotech, Newcastle upon Tyne, UK; 3 Department of Human Genetics, Research Institute for Medical Innovation, Radboud University Medical Center, Nijmegen, The Netherlands; 4 Department of Molecular Biology and Genetics, Aarhus University, Aarhus C, Denmark; 5 Department of Biosciences, Durham University, Durham, UK; 6 Department of Informatics, University of Bergen, Bergen, Norway

**Keywords:** Pluripotent stem cells, RNA

## Abstract

Alternative splicing (AS) is a crucial mechanism contributing to proteomic diversity, which is highly regulated in tissue- and development-specific patterns. Retinal tissue exhibits one of the highest levels of AS. In particular, photoreceptors have a distinctive AS pattern involving the inclusion of microexons not found in other cell types. *PROM1* whose encoded protein Prominin-1 is located in photoreceptor outer segments (OSs), undergoes exon 4 inclusion from the 12^th^ post-conception week of human development through adulthood. Exon 4 skipping in *PROM1* is associated with late-onset mild maculopathy, however its role in photoreceptor maturation and function is unknown. In this study retinal organoids, a valuable model system, were employed in combination with phosphorodiamidate morpholino oligos (PMOs) to assess the role of exon 4 AS in the development of human retina. Retinal organoids were treated with the PMOs for four weeks after which RT-PCR, western blotting and immunofluorescence analysis were performed to assess exon 4 exclusion and its impact on photoreceptors. The transcriptome of treated ROs was studied by bulk RNA-Seq. Our data demonstrate that 55% skipping of *PROM1* exon 4 resulted in decreased Prominin-1 expression by 40%, abnormal accumulation of cones in the basal side of the retinal organoids as well as detectable cone photoreceptor cilium defects. Transcriptomic and western blot analyses revealed decreased expression of cone, inner segment and connecting cilium basal body markers, increased expression of genes associated with stress response and the ubiquitin-proteasome system, and downregulation of autophagy. Importantly, the use of retinal organoids provides a valuable platform to study AS and unravel disease mechanisms in a more physiologically relevant context, opening avenues for further research and potential therapeutic interventions. Together our data indicate that cones may be more sensitive to *PROM1* exon 4 skipping and/or reduced Prominin-1 expression, corroborating the pathogenesis of late-onset mild maculopathy.

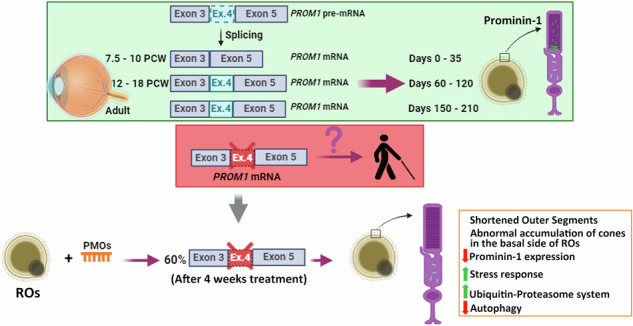

## Introduction

Alternative splicing (AS) makes a significant contribution to gene expression during retinal development and homeostasis. Specifically, mature photoreceptors follow a developmentally regulated splicing program that displays a switch-like pattern with high exon inclusion levels in photoreceptors and almost complete exclusion in extra-retinal tissues [1]. Usually, the alternatively spliced exons are short, ranging from 3 to 27 nucleotides: hence they are referred to as microexons. It has been suggested that retina-enriched microexons (RetMIC) provide a unique proteome specialization necessary for the proper development and functioning of the photoreceptors and their outer segments (OSs), which are highly modified primary cilia.

*PROM1*, a RetMIC containing gene [2] is located on chromosome 4p15.32, comprises 28 exons and encodes Prominin-1 (Uniprot: O43490), a transmembrane glycoprotein of 865 amino acids [2, 3]. Its unique structure consists of an N-terminal extracellular domain (E1), five transmembrane domains with two large and highly glycosylated extracellular loops (E2 and E3) and a C-terminal cytoplasmic domain (I1) [3, 4]. Prominin-1 was originally identified by two independent groups in murine neuroepithelial cells [5] and in human hematopoietic stem and progenitor cells [6]. In addition, Prominin-1 expression extends to several epithelial and non-epithelial cell types, such as photoreceptors and glial cells [7, 8], and several types of malignant tumours [9]. At the subcellular level, Prominin-1 is selectively localized at the plasma membrane protrusions such as microvilli, primary and motile cilia [3, 4]. Within these membrane protrusions, Prominin-1 is associated with a cholesterol-based membrane microdomain [7]. In addition to this selective localization, Prominin-1 is associated with membrane particles (prominosomes) which are released in various body fluids including cerebrospinal fluid and saliva [10, 11].

While the exact function of Prominin-1 remains unclear, its role in maintaining retinal homeostasis is evident, as mutations in *PROM1* have been linked to retinal degeneration in humans with a variety of retinal phenotypes, including autosomal recessive retinitis pigmentosa [12, 13] with macular atrophy [14], autosomal recessive cone-rod dystrophy [15–17], autosomal dominant cone-rod dystrophy and macular phenotypes [18], such as Stargardt-like [19] and bull’s eye maculopathies [20]. In the retina, Prominin-1 is expressed in both photoreceptor and retinal pigment epithelium (RPE) cells, playing an important role in disk morphogenesis and structural stability [3, 21] and regulation of photoreceptor autophagy [22, 23], respectively.

To date, seven Prominin-1 alternative spliced isoforms that differ in the N- and C-terminal encoding exons have been identified in humans, with tissue-specific distribution [4, 7, 14, 24]. Permanyer et al. [14] reported that 98% of the retinal Prominin-1 isoforms contain the exon 4 in the N-terminal domain and 90% of them skip exon 25 in the C-terminal domain. The main difference between the two main isoforms, s11 and s12, is the exclusion or inclusion of exon 24 in the C-terminal domain, respectively. The presence of distinct cytoplasmic C-terminal domains suggests that splicing variants may interact with different cytoplasmic protein partners.

Our group previously demonstrated that *PROM1* displays an altered splicing pattern during human retinal development, consisting of increased exon 4 inclusion (54%) and exon 25 skipping (44.4%) during the 12^th^ - 18^th^ week of human development [25]. While exon 4 skipping seems to occur naturally in extraocular tissue, its absence in the retina has been associated with late-onset mild maculopathy [26]. These facts suggest that exon 4 and its encoded nine amino acids (PETVILGLK) may play an important role in photoreceptor function and/or maintenance.

To study the impact of *PROM1* AS in photoreceptor development, we have used phosphorodiamidate morpholino oligos (PMOs) to modify *PROM1* splicing in retinal organoids (ROs) derived from human induced pluripotent stem cells (hiPSCs). We studied in detail both exon 24 and exon 25 AS during in vivo and in vitro retinogenesis but carried out the PMO treatment only for exon 4 splicing modification because of its involvement in late-onset mild maculopathy [26]. Our data demonstrate that 55% skipping of exon 4 results in decreased Prominin-1 expression by 40%, shortening of photoreceptor OSs and abnormal accumulation of cone photoreceptors in the basal side of the retinal organoids. Transcriptomic and western blot analyses revealed an increased expression of genes associated with the ubiquitin-proteasome system and the stress response, and downregulation of key proteins regulating autophagy. By yeast two-hybrid screening of retinal cDNA libraries, we identified that Prominin-1 potentially interacts with endophilin-A3, which is involved in transport of proteins to membranes with high curvature. Together our data provide significant insights into the role of Prominin-1 during the development of photoreceptors under normal homeostasis and disease conditions.

## Materials and methods

### Human tissue

The MRC/Wellcome Trust-funded Human Developmental Biology Resource (HDBR, www.hdbr.org) provided the human embryonic and foetal ocular material with appropriate maternal written consent and approval from the NHS Health Research Authority’s Newcastle and North Tyneside 1 Research Ethics Committee (REC ref: 08/H0906/21 + 5). Adult human retinal samples were collected with proper consent and approval from NRES Committee Northeast - Newcastle & North Tyneside 1 (REC number: 18/YH/04/20). Neural retina, RPE and non-retinal tissues (cornea, lens and vitreous) were isolated and immediately immersed into RNA*later* (Invitrogen) and stored at −20 °C. Embryonic and foetal retinal tissue was collected from 7.5 to 21 post-conception weeks (PCW) of human development. The RPE and non-retinal tissues were collected from the 9, 12 and 18 PCW of human development. Further information on the samples is provided in Supplementary Table S1.

### RO differentiation

hiPSCs (WT2) [27] and human embryonic stem cells (hESCs) (CRX-GFP H9) [28] were expanded in mTeSR™1 (StemCell Technologies) on growth factor reduced Matrigel coated plates (BD Biosciences) and maintained at 37 °C and 5% CO_2_. Medium was changed daily and hiPSCs and hESCs were checked for mycoplasma routinely. For the generation of ROs, confluent hiPSCs and hESCs were dissociated into single cells using Accutase (Life Technologies) and plated at a density of 7000 cells/well in 96-well lipidure-coated (Amsbio) U-bottom plates (Helena Biosciences) in mTeSR™1 media supplemented with 10 μM Y-27632 (ROCK Inhibitor, Chemdea). After two days, differentiation medium was added consisting of knockout-Dulbecco’s modified Eagle’s medium/ Ham’s F-12 (Life Technologies), 20% KnockOut Serum Replacement (Life Technologies), 2% B-27 (Life Technologies), 1% GlutaMAX (Life Technologies), 1% Pen/Strep, 1% MEM Non-essential Amino Acids (Life Technologies) and 5 ng/ml of recombinant human Insulin-Like Growth Factor 1 (IGF-1, Sigma-Aldrich). Medium was changed every two days [29]. At day 18, the differentiation media was supplemented with 10% Fetal Bovine Serum (Gibco®/Life Technologies), Taurine (0.1 mM; Merck) and T3 (40 ng/ml; Merck) and changed every 2-3 days. On day 30 of differentiation, media was supplemented with 1% N-2 Supplement (Gibco®/Life Technologies), 1% chemically defined lipid concentrate (Life Technologies), and IGF-1 concentration was increased from 5 to 10 ng/ml. From day 90, ROs were further cultured in 6-well low attachment plates (Corning) and Retinoic Acid (1 µM; Merck) was added to the media until day 120 of differentiation. ROs were collected at different stages of differentiation: days 35, 60, 90, 120, 150, 180 and 210 for RT-PCR, western blot, and immunofluorescence analyses. In addition, “eye-like” ROs were collected at days 35, 60 and 90 of differentiation for RT-PCR analysis. The differentiation was repeated at least three times for each set of experiments.

### RNA isolation and cDNA conversion

RNA isolation was performed using the ReliaPrep™ RNA Cell/Tissue Miniprep System kits (Promega), following manufacturer’s instructions. Three biological replicates were collected per time point, with a total of 12 ROs in each. Extracted RNA was further purified using TURBO DNA-*free*™ Kit (Thermo Fisher Scientific) and measured with Qubit® RNA BR Assay Kit (Life Technologies). One microgram of RNA was reverse transcribed into cDNA with random primers using GoScript™ Reverse Transcription System (Promega).

### Reverse transcription-polymerase chain reaction (RT-PCR)

To assess *PROM1* AS, specific pair of primers were designed using several websites (NCBI, Primer3 and UCSC genome) (Sigma-Aldrich; Supplementary Table S1). For the PCR reaction, cDNA was amplified using GoTaq® G2 DNA Polymerase (Promega). The PCR consisted of an initial denaturation at 95 °C for 5 min, followed by a 35-cycle programme of 95 °C for 20 s, 60 °C for 30 s, 72 °C for 30 s, and a final extension at 72 °C for 5 min. PCR was carried out using a Mastercycler® thermal cycler. All the PCR products were resolved on 2% agarose gel mixed with GelRed® Nucleic Acid Stain (Biotium). The images were captured using the GelDoc-It® 310 Imaging System (UVP) and the VisionWork®LS software. Densitometric analyses from agarose gels were performed using Fiji software (ImageJ version 2.0.0).

### Quantitative real-time polymerase chain reaction (qRT-PCR)

Relative gene expression was determined by qRT-PCR using GoTaq® qPCR Master Mix (Promega). C_t_ values were measured utilizing QuantStudio™ 7 Flex Real-Time PCR System (Thermo Fisher Scientific). Primers against *GAPDH* were used as the endogenous control for sample normalization. Primer sequences are shown in Supplementary Table S1. At least three technical replicates were included per each biological replicate.

### Immunofluorescence analysis

ROs were collected and prepared for embedding following the protocol described by Corral-Serrano et al. [30] (*n* = 5 ROs/condition/biological triplicate). Briefly, ROs were washed once in PBS and fixed in 4% PFA + 5% sucrose solution for 30 min. ROs were then placed in 6.25% sucrose for 1 h, followed by 12.5% sucrose for 1 h and in 25% sucrose overnight. All incubation steps were performed at 4 °C. ROs were then embedded in Optical Cutting medium (OCT; CellPath), frozen and cryosectioned (10 µm thick).

For immunohistochemistry, sections were washed several times in PBS and blocked with 10% goat serum (Thermo Fisher Scientific), 0.3% Triton X-100 (Sigma-Aldrich) in PBS for 1 h. Slides were incubated with the appropriate primary antibody overnight at 4 °C (Supplementary Table S1). Sections were then washed in PBS several times and incubated with the secondary antibody for 2 h at room temperature in the dark. Alexa Fluor 488 and 546 secondary antibodies (Thermo Fisher Scientific) were used at a 1:1000 dilution. Nuclei were visualized using Hoechst (1:1000, Thermo Fisher Scientific) and Vectashield (2B Scientific) was used as mounting medium. Images were captured using Zeiss AxioImager microscope with Apotome (Zeiss, Germany) using 20x and 63x objectives. Approximately 5 images were taken per antibody combination, imaging ROs generated from different differentiations. Final images are presented as a maximum projection and adjusted for brightness and contrast in Adobe Photoshop (Adobe Systems).

Image quantitation was performed as described by Dorgau et al. [29] using the MATLAB software (Mathworks). Data were plotted and statistically analysed using Prism version 10.1.2 (GraphPad software). Bright-field images were captured with an AxioVert microscope (Zeiss, Germany) using 5x objective.

### Outer segments length and cilia frequency measurements

Images were obtained using Zeiss AxioImager microscope with Apotome using 63x oil objective. Maximum intensity projections of z-stacks were used for the analysis. Manual counting was performed to measure photoreceptor OSs length, cilia length and cilia incidence. Photoreceptor OSs length was measured using ImageJ by quantifying the length from the outer limiting membrane defined by CRALBP immunostaining to the distal edge of the OS-like structures defined by PRPH2 immunostaining. Connecting cilia length was measured by quantifying the length of each Arl13b immunopositive axoneme emerging from pericentrin-positive basal bodies. A minimum of 350 cilia from at least five different ROs were analysed per biological replicate. Nuclei were counted manually, and data was then used to calculate ciliary incidence. Statistical significance was analysed by one-way ANOVA using GraphPad Prism version 10.1.2.

### Scanning electron microscopy (SEM)

ROs obtained from 4 weeks-several doses of PMO treatment (*n* = 3 ROs per treated group) were fixed in 2% glutaraldehyde in 0.0033 M Sorensen’s Phosphate Buffer pH 7.3 (TAAB Laboratories Equipment Ltd) for at least 24 h. After several washing steps in the mentioned buffer, an ethanol dehydration process was performed, starting with 2%, 50%, and 75% ethanol for 30 min followed by twice 100% ethanol incubations for 1 h each. The final dehydration step was conducted using carbon dioxide in a Baltec Critical Point Dryer (Leica Geosystems Ltd). Afterwards, ROs were mounted on an aluminium stub with Achesons Silver ElectroDag (Agar Scientific) and left to air-dry overnight. Subsequently, a gold coating, ranging from 5 to 10 nm in thickness, was applied to the ROs utilizing a Polaron SEM Coating Unit (Quorum Technologies Ltd) and imaged using a Tescan Vega LMU Scanning Electron Microscope with a Tescan supplied software (Tescan).

### Western blot analysis

For western blot analysis, three biological replicates were collected per time point or per condition, with a total of 12 ROs in each. Then, ROs were washed with cold PBS, re-suspended in lysis buffer (PhosphoSafe Extraction Reagent; Sigma-Aldrich) and lysed by ultrasonication (x3, for 5 s) (Misonix Sonicator 3000) to obtain whole cell lysate. The protein concentration was determined using the BCA Protein Assay kit (Thermo Fisher Scientific). Twenty μg of protein extract was loaded on Bolt™ Bis-Tris Plus Mini Protein gels, 4-12% (Invitrogen) and transferred to PVDF transfer membrane (Invitrogen). The membrane was blocked with 5% dried skimmed milk in 1× Tris Buffered Saline (Santa Cruz Biotechnology)—0.1% Tween 20 (Bio-Rad) (TBST) for 1 h at room temperature and incubated with primary antibodies (1:500; Supplementary Table S1) overnight at 4 °C. After three TBST washes, the membrane was incubated with secondary antibodies (1:2000) for 1 h at room temperature and again washed three times with TBST. Membranes were revealed using SuperSignal™ West Pico Plus Chemiluminescent Substrate Kit (Thermo Fisher Scientific) and imaged on Amersham Imager 600, followed by densitometric analysis. Values were normalized against the GAPDH housekeeping control. Detailed information on the Prominin-1 antibodies used for western blot analysis is shown in Supplementary Table S1.

### AlphaFold modelling of protein isoforms

Prominin-1 isoforms structures were modelled using a local installation of AlphaFold v2.3.2 [31, 32] and using *Homo sapiens* Prominin-1 sequences (Uniprot: O43490). All figures of protein structures were prepared using PyMOL v. 2.5 (Schrodinger LLC, https://pymol.org).

### Phosphorodiamidate Morpholino Oligos treatment

hiPSC-ROs were cultured in low attachment 24 or 6-well-plates for the PMO screening. *PROM1*-specific and standard Control PMOs (Supplementary Table S1) were purchased from Gene Tools. PMO stock solutions (1 mM) were added at the desired concentration to fresh culture media supplemented with 6 μM Endo-Porter polyethylene glycol (GeneTools). For the experiment utilizing several doses’ treatment, ROs were treated with PROM1-PMO or Ctrl-PMO every week for 4 weeks. Each treatment was repeated three times independently.

### LDH cytotoxicity assay

To determine the cytotoxic effect of the PMOs on ROs, the Pierce LDH Cytotoxicity Assay Kit (Fisher Scientific) was used according to the manufacturer’s manual. All conditions were set out in triplicates. The absorbance was measured at 490 nm and 680 nm using a Varioskan LUX Multimode Microplate Reader (Thermo Fisher Scientific). Excel and GraphPad Prism v.10.1.2 were used for further statistical analysis.

### Bulk RNA-Seq

ROs were collected after 4 weeks several-doses treatment, with either PROM1-PMO or Ctrl-PMO, for RNA extraction. Three biological replicates were used per condition, each of which had ten ROs. Samples were processed for sequencing using the Illumina Stranded mRNA Library Prep Kit (Illumina) following manufacturer’s instructions. Libraries were sequenced (~50 million 75 bp single end reads per sample) on an Illumina NextSeq 500 (75 cycle High Output v2 kit).

Reads were quality checked using FastQC and MultiQC. Reads were quantified by Salmon software (version 1.9.0) [33] using the Human (GRCh38) transcriptome from Gencode (release version 42). The resulting count tables were imported into R (version 4.2.2) with tximport (version 1.26.1), and differential expression analysis was conducted with DESeq2 (version 1.38.2) [34]. Significant DEGs were identified by absolute value of log2(FoldChange) >= log (1.5) = 0.5849625 & adjusted *p*-value <= 0.05 (Supplementary Table S1). To obtain the enriched ontology clusters, upregulated and downregulated genes were collected and analysed using Metascape [35]. A network plot with a subset of enriched terms was created (where terms with a similarity >0.3 were connected by edges) and visualized using Cytoscape v3.10.1.

### Chymotrypsin-like proteasome activity assay

The chymotrypsin-like protease activity was quantified in the ROs after 4 weeks-several doses of PMO treatment (*n* = 20 ROs per treated group per biological replicate) using the Proteasome Activity Assay Kit (Abcam) following manufacturer’s protocol. Protease activity was measured by the fluorescence intensity (Excitation/Emission = 350/440 nm) using a Varioskan LUX Multimode Microplate Reader and normalized to the protein amount.

### Yeast two-hybrid assay

For the yeast two-hybrid assay, four different Prominin-1 fragments (E1, E2, E3 and I1) were cloned into a pBD vector containing the binding domain of the *GAL4* transcription factor (Gateway cloning, Life Technologies). The pBD vectors served as bait in screening both human and bovine retinal cDNA libraries (generated using oligo-dT and random primers, respectively) [36].

To assess interactions, activation of reporter gene (*HIS3* and *ADE2*) was analysed via growth on selective media together with α-galactosidase colorimetric assay (*MEL1* reporter gene). cDNA inserts of clones suspected to encode interaction partners were subsequently characterized through Sanger sequencing.

### Statistical analysis

The sample size was chosen based on previous experiments performed by our group. In all cases at least three biological replicates were included, with sufficient organoid numbers or RNA/protein quantity to generate high quality statistically significant results. All statistical tests were conducted with GraphPad Prism v.10.1.2. Data were plotted as mean values with error bars representing standard error of the mean (SEM). Additional information such as *n* values (the number of independent biological replicates) is explained in figure legends. Data were tested for normality using a variety of tests provided by GraphPad Prism software. F tests assessing variance between experimental groups were performed with the GraphPad Prism software. This was followed by the calculation of standard deviation and standard error of the mean, with the latter being shown in all graphs presented in this study. Statistical significance between two groups was performed using unpaired *t*-test (two-tailed). For comparison of multiple groups, one-way ANOVA followed by Sídák’s multiple comparisons test was used. Statistical significance of pair-wise comparisons is indicated by asterisks as follows: *p < 0.05, **p < 0.01, ***p < 0.001, and ****p < 0.0001. Significance was defined as a *p*-value < 0.05.

## Results

### *PROM1* AS during human retinal development

AS is a widely used mechanism used by many cell types to generate protein isoforms that are necessary for their function. The photoreceptors are characterised by a specific splicing program that is in part driven by Musashi I. This displays a switch-like pattern with high exon inclusion levels in photoreceptors and almost complete exclusion outside the retina [37]. In addition, specialization of the photoreceptor transcriptome by *Srrm3*-dependent microexons is required for OSs maintenance and vision [2]. In our previous study, we discovered that transition from foetal to adult retina was characterised by a large increase in the percentage of mutually exclusive exons that code for proteins involved in photoreceptor maintenance. Of those, *PROM1*, a gene involved in photoreceptor disks morphogenesis in the retina, was shown to undergo exon 4 inclusion and exon 24/25 skipping [25].

**Fig. 1:**
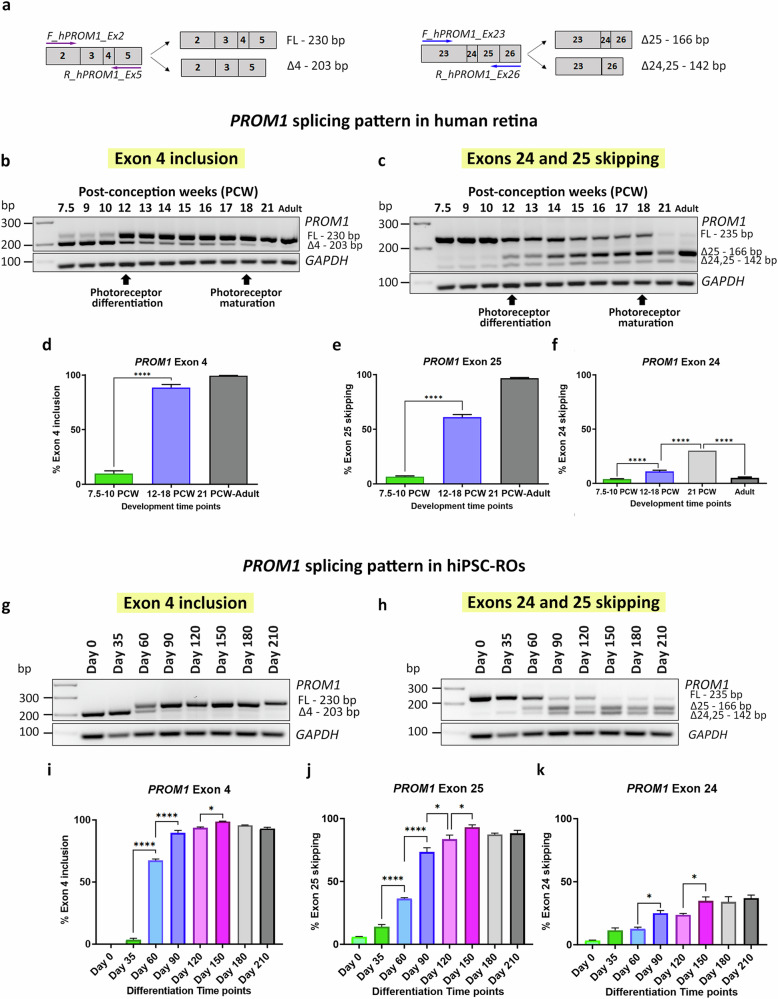
*PROM1* alternative splicing in retina in vivo and in vitro. **a** Schematic representation of the primers used to amplify exon 4 (on the left) and exons 24 and 25 (on the right). **b**–**f** RT-PCR analysis of *PROM1* splicing during human retinal development: **b** Exon 4 *PROM1* and **c** Exon 25 *PROM1* splicing pattern; **d**–**f** Quantification of exon 4 inclusion, exon 25 skipping and exon 24 skipping. Data are shown as mean ± SEM (*n* = 3–7). Statistical significance was assessed using one-way ANOVA with Sídák’s post hoc test. ****p < 0.0001. **g**–**k** RT-PCR analysis of *PROM1* splicing during hiPSC-ROs differentiation: **g** Exon 4 *PROM1* and **h** Exon 25 *PROM1* splicing pattern; **i**–**k** Quantification of exon 4 inclusion, exon 25 skipping and exon 24 skipping. Data are shown as mean ± SEM (*n* = 3). Statistical significance was assessed using one-way ANOVA with Sídák’s post hoc test. *p < 0.05, ****p < 0.0001. FL Full-length product, △4 Exon 4 skipped, △25 Exon 25 skipped, △24,25 Exons 24 and 25 skipped, PCW Post-conception week.

To validate the previous results and assess in detail the dynamics of *PROM1* AS, RT-PCR was performed using specific primers that amplified the alternative exons of interest, exon 4 and exon 25 (Fig. 1a) followed by Sanger sequencing of PCR products (Supplementary Fig. S1a, b). Both human embryonic/foetal retina (7.5-21 PCW) and adult retina samples were used. In total, 41 samples were analysed, using at least three biological replicates per developmental stage, except from 18 PCW and 21 PCW time points due to the scarcity of the tissue. This analysis demonstrated a significant increase in exon 4 inclusion from 12 PCW (Fig. 1b), which is maintained in the adult retina. For exon 25, three different PCR products were detected from 12 PCW, demonstrating skipping of exon 25 as well as 24 and 25 (Fig. 1c). Semiquantitative analysis showed that exon 4 inclusion increased to 88% at 12–18 PCW and reached 99% in the adult stage (Fig. 1d). The exon 25 skipping was more gradual compared to exon 4 inclusion, increasing to 61% at 12–18 PCW and reaching 97% in the adult retina (Fig. 1e). In contrast, exon 24 skipping was much lower than exon 25 skipping and increased from 12 to 18 PCW reaching its maximum at 21 PCW and then decreased at the adult stage (Fig. 1f).

### In vitro ROs recapitulate *PROM1* AS pattern in vivo

Pluripotent stem cell -derived ROs have been shown to recapitulate retinal development and were used as a tool to investigate *PROM1* AS in vitro. ROs derived from both hiPSCs and hESCs were generated using a previously established protocol [29] and collected at different time points of differentiation, from day 0 to 210. In total, eight time points were analysed, using three biological replicates per time point. Our RT-PCR analysis showed that exon 4 inclusion increased significantly at day 60 and 90 of differentiation in hiPSC- and hESC-ROs, respectively (Fig. 1g, i; Supplementary Fig. S1c, e), reaching maximum levels at day 150 of differentiation. *PROM1* transcripts lacking exon 4 were present until day 60 of RO differentiation with *PROM1* containing exon 4 transcripts being solely expressed from day 90 until the end of the differentiation process. Exons 24 and 25 were both included in *PROM1* transcripts in the undifferentiated pluripotent stem cells. Exon 25 skipping started from day 35 and increased alongside the differentiation process, reaching maximal levels at day 150 of differentiation (Fig. 1h, j; Supplementary Fig. S1d, f).

Exon 24 skipping increased during RO development and remained stable from day 150 until the end of differentiation (Fig. 1k). In case of hESC-ROs, there were some variations, but the overall trend was an increase from day 0 until the end of differentiation process at day 210 (Supplementary Fig. S1g). Together these data demonstrate that AS in vivo can be recapitulated in vitro, establishing ROs as a useful tool to study *PROM1* exon skipping and inclusion.

### *PROM1* splicing pattern in the eye is cell type-specific

Previous studies have shown natural exon 4 skipping in extraocular tissues such as saliva, blood, or fibroblasts [26] but did not address *PROM1* AS in RPE or non-retinal tissues. To gain more insight about this phenomenon, *PROM1* splicing was studied in RPE, cornea, lens and vitreous. Different stages of development were assessed namely 9, 12 and 18 PCW as well as the adult stage, using three biological replicates per time point.

**Fig. 2:**
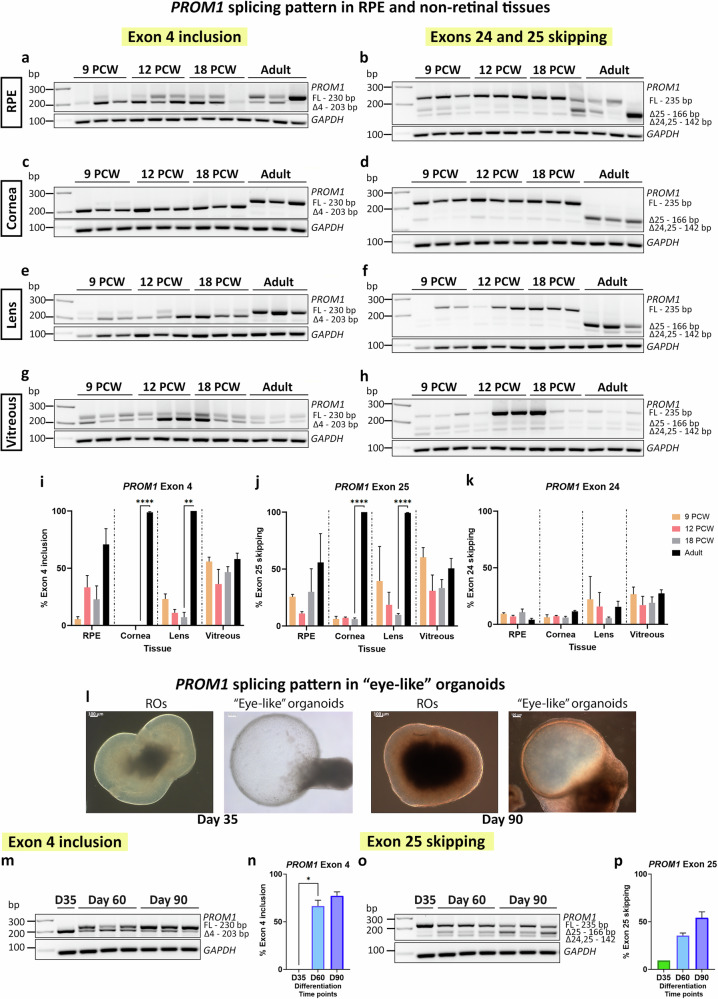
*PROM1* alternative splicing in RPE and non-retinal tissues. **a**–**k** RT-PCR analysis of *PROM1* splicing in RPE and non-retinal samples: (**a**, **b**) RPE; (**c**, **d**) cornea; (**e**, **f**) lens and (**g**, **h**) vitreous; (**i**–**k**) Quantification of exon 4 inclusion, exon 25 skipping and exon 24 skipping. Data are shown as mean ± SEM (*n* = 3). Statistical significance was analysed by one-way ANOVA with Sídák’s post hoc test. **p < 0.01, ****p < 0.0001. **l**–**p** Study of *PROM1* splicing in “eye-like” ROs. **l** Representative bright**-**field images showing the morphology of ROs and “eye-like” organoids at days 35 and 90 of differentiation. Scale bars: 100 μm. **m**–**p** RT-PCR analysis of *PROM1* splicing in “eye-like” ROs at three different time points of differentiation: **m** Exon 4 *PROM1* splicing pattern; **o** Exon 25 *PROM1* splicing pattern; **n**, **p** Quantification of exon 4 inclusion and exon 25 skipping. Data are shown as mean ± SEM (*n* = 3). Statistical sig*n*ificance was analysed by one way-ANOVA with Sídák’s post hoc test. *p < 0.05. FL Full-length product, △4 Exon 4 skipped, △25 Exon 25 skipped, △24,25 Exons 24, 25 skipped, PCW Post-conception week.

In the RPE, exon 4 inclusion is apparent from 12 PCW, although the *PROM1* transcripts without exon 4 are more abundant (Fig. 2a). Both the cornea and lens show a remarkably similar pattern, with almost 100% of exon 4 exclusion up to 18 PCW of development and 100% inclusion at the adult stage (Fig. 2c, e). In the case of the vitreous, there is the same amount of *PROM1* transcripts with or without exon 4, except at 12 PCW, where exon 4 is excluded in ca. 60% of the transcripts (Fig. 2g). Concerning exon 25, the majority of *PROM1* transcripts (except the vitreous) include both exons 24 and 25 during foetal development. Exon 25 skipping increases in the adult stage, with 55% of skipping in the RPE and 100% for both cornea and lens tissues. *PROM1* exon 24 skipping is low but stable throughout development and adulthood for all tissues, with the RPE and cornea displaying lower levels of skipping compared to the lens and vitreous (Fig. 2b, d, f, h, j, k).

During the PSC differentiation, we observed the presence of “eye-like” ROs, which in addition to neural retina also contained conjunctival epithelium, limbal-corneal epithelium, and corneal stroma cells [38]. *PROM1* splicing in these more complex ROs nicely recapitulated the splicing patterns observed during retinal development in vitro and in vivo, with exon 4 inclusion and exon 25 skipping increasing until day 90 of differentiation, although the inclusion/exclusion levels reached were lower compared to RO differentiation (Fig. 2l–p). These “eye-like” ROs are not evident after day 90, most likely due to culture conditions being optimised for neural retina differentiation.

### Prominin-1 is localized to the photoreceptor OSs

**Fig. 3:**
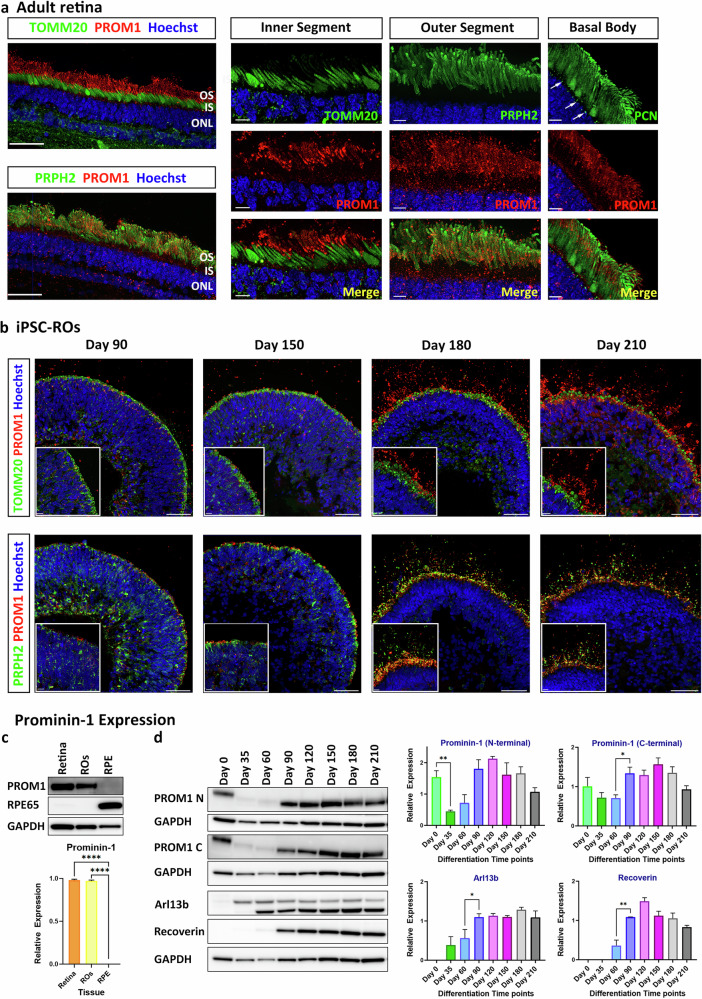
Prominin-1 expression in adult retina and hiPSC-ROs. **a** Immunofluorescence analysis of Prominin-1 in adult retina. Two different Prominin-1 antibodies were used: 1^st^ Extracellular loop (E2) Prominin-1 (PROM1, red) antibody in combination with either TOMM20 (green) marking the ISs or PCN marking the basal body of the connecting cilium (white arrows); N-terminal Prominin-1 (PROM1, red) antibody in combination with PRPH2 (green) marking the OSs. **b** Immunofluorescence analysis of Prominin-1 (PROM1, red) during ROs differentiation in combination with TOMM20 (green) and PRPH2 (green) antibodies. **a**, **b** Cell nuclei were stained with Hoechst. Scale bars: 50 μm; 10 μm (magnification). **c** Representative western blot and quantification analysis showing Prominin-1 expression in human adult retina and RPE samples and ROs at day 180 of differentiation. RPE65 expression was also assessed. GAPDH was used as a loading control. Data represent the mean ± SEM (*n* = 3). Statistical significance was analysed by one-way ANOVA with Sídák’s post hoc test. ****p < 0.0001. **d** Representative western blot and quantification analysis showing Prominin-1 expression during ROs differentiation. Both the Prominin-1 N-terminal and C-terminal antibodies were used. Arl13b and recoverin expression were also assessed. GAPDH was used as a loading control. Data represent the mean ± SEM (*n* = 3). Statistical significance was analysed by one-way ANOVA with Sídák’s post hoc test. *p < 0.05, **p < 0.01. IS inner segment, ONL outer nuclear layer, OS outer segment, PCN pericentrin, PRPH2 peripherin-2.

To determine the localization of Prominin-1, immunohistochemistry was performed in the adult human retina. The following antibodies were used: Translocase of Outer Mitochondrial Membrane 20 (TOMM20) to mark the ellipsoid region of the photoreceptor inner segments (ISs), peripherin-2 (PRPH2) to mark the photoreceptor OSs and pericentrin (PCN) to mark the basal body of the photoreceptors. This analysis showed that Prominin-1 is localized in the photoreceptor OSs, colocalizing with PRPH2 but not TOMM20 or PCN (Fig. 3a).

To assess Prominin-1 expression throughout RO development, immunohistochemistry and western blot analyses were performed at various time points of differentiation (Fig. 3b, c). At the early stages of differentiation (day 35 and 60), Prominin-1 is barely present (Supplementary Fig. S2), but by day 90, Prominin-1 starts to localize at the apical site of the ROs, colocalizing with Arl13b, a connecting cilia marker (Supplementary Fig. S2). From day 180, Prominin-1 colocalizes with PRPH2 in the photoreceptor OSs, but not TOMM20 (Fig. 3b) or CRALBP (Supplementary Fig. S2), which mark the ellipsoid and myoid regions of the ISs, respectively. The same pattern was observed at day 210 ROs, with restricted localization of Prominin-1 to the OSs, recapitulating the pattern observed in the adult retina (Fig. 3a).

Western blot analysis demonstrated strong expression in neural retina in adult and day 180 ROs (Fig. 3c) but not in the RPE layer. Two different Prominin-1 antibodies were used in the WB analysis of ROs along the differentiation process. N-terminal Prominin-1 antibody was raised against amino acids 20–108 of Prominin-1, recognizing the sequence encoded by exons 2–5, whereas C-terminal Prominin-1 antibody was raised against amino acids 841–865, recognizing the sequence encoded by exons 26–27 (Supplementary Table S1). With both antibodies we were able to demonstrate very low expression at the early stages of differentiation (day 35 and day 60) and a distinct increase in Prominin-1 expression from day 90, which was maintained until the last time point of differentiation, day 210 (Fig. 3d), corroborating the immunohistochemistry results.

### Phosphorodiamidate morpholino oligos (PMOs) screening

Because exon 4 is present in >97% of the isoforms in the retina and its skipping results in inherited retinal disease [26], we hypothesised that exon 4 could play an important role in photoreceptor development, function and/or maintenance. Given the unavailability of retinal tissue from the patients, we used PMOs to modify exon 4 splicing during RO differentiation.

Two PMOs were designed to cause exon 4 skipping with the first targeting the splice acceptor site between intron 3 and exon 4 of *PROM1* (PMO1), and the second targeting the splice donor site between exon 4 and intron 4 (PMO2) (Supplementary Table S1). Both PMOs were evaluated in hiPSC-ROs at day 90 of differentiation, alongside a standard Control-PMO (Ctrl-PMO) (Supplementary Fig. S3a). Exon skipping (ES) efficiency was estimated according to the percentage of exon-skipped products, relative to total products [skipped product/ (full-length + skipped product)]. RT-PCR analysis revealed that ES efficiency was low after 48 h at 10 μM PMO dose in all the treated groups (Supplementary Fig. S3b, c). Higher concentrations of PMOs (25 μM, 50 μM and 75 μM) were investigated, showing a dose-dependent exon skipping in the case of PMO1 treatment, however no differences were observed between the PMO2 and Ctrl-PMO treated groups (Supplementary Fig. S3d, e). As the ES efficiency was still not high enough, higher concentrations of PMOs were assessed (100 μM, 200 μM and 250 μM). In this case, RT-PCR analysis showed a higher proportion of exon 4 skipped product-band, suggesting higher ES efficiency, with PMO1 being more efficient than PMO2 (Supplementary Fig. S3f, g). Nonetheless, a dose dependent increase in toxicity was observed from 25 µM and higher, for this reason 10 μM was chosen as the most optimal concentration to cause exon skipping without inducing toxicity in the ROs (Supplementary Fig. S3h).

### Effect of exon 4 skipping on *PROM1* expression

**Fig. 4:**
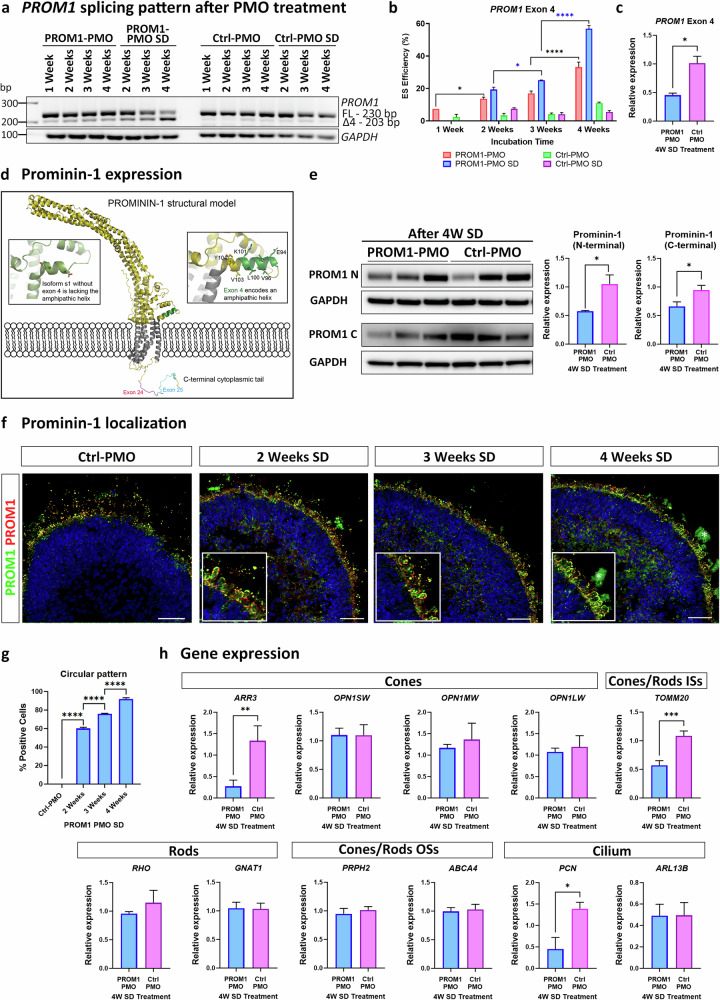
Treatment of hiPSC-ROs at day 180 of differentiation with PROM1-PMO or Ctrl-PMO from 1 to 4 weeks. **a**, **b** RT-PCR analysis and quantification of exon 4 skipping efficiency after PMO treatment. Data are shown as mean ± SEM (*n* = 3). Statistical significance was assessed using one-way ANOVA with Sídák’s post hoc test. *p < 0.05, ****p < 0.0001. **c** qRT-PCR analysis showing downregulation in expression of *PROM1* transcript with exon 4 after PROM1-PMO treatment. Data are shown as mean ± SEM (*n* = 3). Statistical sig*n*ificance was assessed using unpaired Student *t* test. *p < 0.05. **d** AlphaFold predicted model of Prominin-1 structure. Zoom in on exon 4 (s2 isoform) shows that part of the helix in green which is missing in s1 and connects to the following helix via a loop. **e** Representative western blot and quantification analysis showing decreased level of Prominin-1 after PROM1-PMO treatment. Both the Prominin-1 N-terminal and C-terminal antibodies were used. GAPDH was used as a loading control. Data are shown as mean ± SEM (*n* = 3). Statistical significance was assessed using unpaired Student *t* test. *p < 0.05. **f** Representative images of ROs treated with Ctrl-PMO for 4 weeks with several doses or with PROM1-PMO from 2 to 4 weeks with several doses. Prominin-1 N-terminal (PROM1, green) and E2 (PROM1, red) antibodies were used. Higher magnification images revealed the circular pattern of Prominin-1 around the ISs after the treatment (*marks background signal). Cell nuclei were stained with Hoechst. Scale bars: 50 μm. **g** Quantification of circular pattern. Data are shown as mean ± SEM (*n* = 5 ROs/condition/biological triplicate). Statistical significance was analysed by one-way ANOVA with Sídák’s post hoc test. ****p < 0.0001. **h** qRT-PCR analysis of cones and rod, ISs, OSs and cilia specific markers after several doses of PROM1-PMO treatment for 4 weeks. Data are shown as mean ± SEM (*n* = 3). Statistical significance was determined using u*n*paired Student *t*-test. *p < 0.05, **p < 0.01, ***p < 0.001. FL Full-length product, △4 Exon 4 skipped, OSs outer segments, ISs inner segments, SD Several Doses, UTC untreated.

To increase ES efficiency without causing toxicity, ROs were treated at day 180 of differentiation with 10 μM of Ctrl- or PROM1-PMO with either one dose or several doses from 1 to 4 weeks (Supplementary Fig. S3i). RT-PCR analysis showed that PROM1-PMO treatment caused exon 4 skipping, which increased both with time and the number of doses, reaching 33% after 4 weeks with a single dose and 57% after four doses (Fig. 4a, b). qRT-PCR analysis confirmed that the level of *PROM1* transcripts with exon 4 was reduced by 55% after the 4 weeks-several doses treatment (Fig. 4c).

To predict the structure of the different Prominin-1 isoforms and the impact of exon 4 skipping, AlphaFold modelling was used (Fig. 4d) [31]. All the splice isoforms displayed similar overall structure with 5 transmembrane helices and a large extracellular extension. The large extracellular extension, often referred to as E2 and E3 [3], is predicted to be α-helical and protrudes 15 nm into the extracellular space. Whereas exons 24 and 25 are both found on the intracellular side in the C-terminal tail, exon 4 is predicted to fold into an amphipathic helix (coloured green in Fig. 4d) and is located on the extracellular side close to the position of the first transmembrane helix. This amphipathic helix is lost when exon 4 is skipped (Fig. 4d), which may potentially alter the position of the extracellular extension or the membrane-binding properties of Prominin-1. Although, this isoform, known as s1, lacks the exon 4 which encodes the internal segment of nine amino acids near the N-terminal, its structure is stable as the nine amino acids are in frame. To further assess the impact of exon 4 skipping on expression of Prominin-1, western blotting was carried out with two different antibodies targeting the N-terminal and the C-terminal domains, respectively. Both Prominin-1 antibodies showed a downregulation of Prominin-1 expression after the 4 weeks-several doses PROM1-PMO treatment compared with the Ctrl-PMO (Fig. 4e).

Immunofluorescence analysis was also performed to examine Prominin-1 localization. Two different Prominin-1 antibodies were used, the mentioned N-terminal Prominin-1 antibody and E2 Prominin-1 antibody, which was raised against amino acids 218-324 of Prominin-1, recognizing the region between exons 7-9. In Ctrl-PMO treated ROs, Prominin-1 was located in the OSs of the photoreceptors, whereas in the PROM1-PMO treated ROs, Prominin-1 changed its expression pattern and showed a characteristic circular pattern around the ISs, more noticeable after 4 weeks-several doses PROM1-PMO treatment (Fig. 4f, g). qRT-PCR demonstrated that PROM1-PMO treatment did not affect the expression of genes marking mature cones (*OPN1SW*, *OPN1MW*, *OPN1LW*) and rods (*RHO*, *GNAT1*) or photoreceptor OSs (*PRPH2* and *ABCA4)*. However, we noticed a significant downregulation of the cone precursor marker *ARR3*, the mitochondria-rich ISs gene *TOMM2O* and basal body marker *PCN* in PROM1-PMO treated ROs compared to the control ROs (Fig. 4g).

### PROM1 exon 4 skipping results in photoreceptor cilium defects

**Fig. 5:**
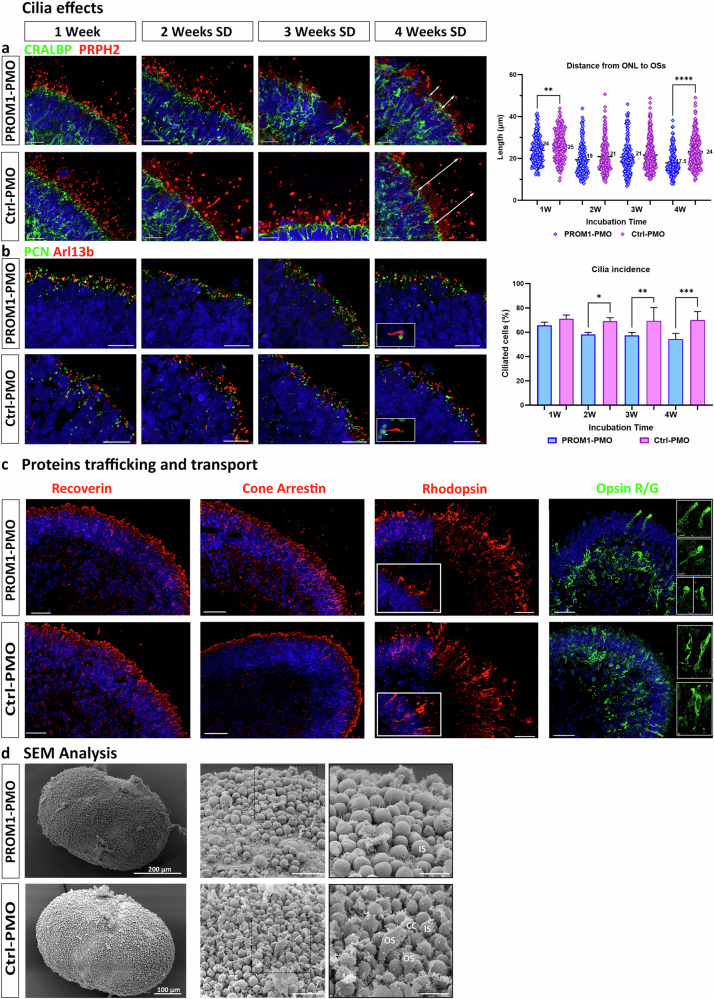
*PROM1* exon 4 skipping effects in cilia and proteins trafficking and transport. **a** Representative images of photoreceptors and the OSs after 1 week to 4 weeks of incubation with either PROM1-PMO or Ctrl-PMO. CRALBP antibody (green) was used to mark both the ellipsoid and myoid regions of the ISs and PRPH2 antibody (red) to mark the OSs. Quantification of ONL to OS distance (from CRALBP staining to the tip of PRPH2 staining, as exemplified by the white arrows) is shown on the right, with the average of each condition displayed in the graph. Data are shown as mean ± SEM (*n* = 3). Statistical significance was analysed by one-way ANOVA with Sídák’s post hoc test. **p < 0.01, ****p < 0.0001. **b** Representative images of connecting cilium using pericentrin (PCN; green) marking the basal body, and Arl13b (red) marking the axoneme. Inset shows cilium structure. Quantification of ciliation of cells is shown on the right. Data are shown as mean ± SEM (*n* = 3). Statistical sig*n*ificance was analysed by one-way ANOVA with Sídák’s post hoc test. *p < 0.05, **p < 0.01, ***p < 0.001. **a**, **b** Cell nuclei were stained with Hoechst. Scale bars: 20 μm. **c** Localization of photoreceptor markers in ROs after PROM1/Ctrl-PMO treatment for 4 weeks with several doses. Antibodies were used against recoverin (red); cone arrestin (red); rhodopsin (red) and opsin R/G (green). Higher magnification images showed rods and cones morphology, revealing a bulbous structure in the case of cones after PROM1-PMO treatment. Representative images from 5 ROs/condition/biological triplicate are shown. Cell nuclei were stained with Hoechst. Scale bars: 50 μm; 10 μm (magnification). **d** Scanning electron microscopy illustrating photoreceptor IS, connecting cilium (CC) and developing OS in ROs after PMO treatment. Scale bars: 20 μm; 10 μm (magnification, black edges images). CC connecting cilium, IS inner segment, OS outer segment, PCN pericentrin, PRPH2 peripherin-2.

Four weeks treatment with several doses of PROM1-PMO resulted in 57% of ES efficiency (Fig. 4a), which was considered high enough to evaluate the impact of *PROM1* mis-splicing in photoreceptors. As *PROM1* is a cilia-related gene, effects in cilia were studied using immunofluorescence analysis. Photoreceptor OSs formation was confirmed in both groups of treated ROs by the presence of PRPH2^+^ structures located apically to the ISs (Fig. 5a). In addition, the length of the segments, from the outer limiting membrane (defined by the CRALBP staining) to the distal edge of the PRPH2^+^ OS-like structures, was measured. No significant changes were observed until the four weeks treatment, with shorter OSs observed in the PROM1-PMO treated ROs compared to the control treated group (Fig. 5a). PROM1-PMO treatment also influenced ciliation levels, with a significantly reduced cilia incidence (measured by Arl13b staining) in the treated ROs compared to the control (Fig. 5b), although in this case, the length of the connecting cilium was similar (data not shown).

Since the connecting cilium is crucial for protein trafficking, immunofluorescence analysis was performed to assess the impact of the decreased number of ciliated cells on the distribution of several proteins associated with mature photoreceptors. Both recoverin and cone arrestin were abundant across the apical layer of the PROM1-PMO treated ROs. Immunostaining with R/G cone opsin revealed a disorganized distribution of cone photoreceptors, with abnormal accumulation in the basal side of the PROM1-PMO treated ROs (76.6% ± 1.3) compared to the control PMO treated ROs (28.9% ± 1.3) (*n* = 5 ROs/treated group/biological triplicate). In addition, the few cone photoreceptors situated in the apical aspect of the organoid presented an abnormal bulbous cilia morphology (Fig. 5c). No obvious abnormalities were observed for Rhodopsin localization although the fraction of rods was decreased in the PROM1-PMO treated ROs compared to the control (25% ± 1.4 vs. 34.3% ± 2.6, respectively; **p < 0.005) (*n* = 5 ROs/treated group/ biological triplicate). Scanning electron microscopy demonstrated the presence of photoreceptor connecting cilium and ISs in both groups, however a higher number of OSs were captured in the control group, confirming the immunohistochemistry results (Fig. 5d).

### Bulk RNA-Seq analysis suggests downregulation of autophagy in response to *PROM1* exon 4 skipping

**Fig. 6:**
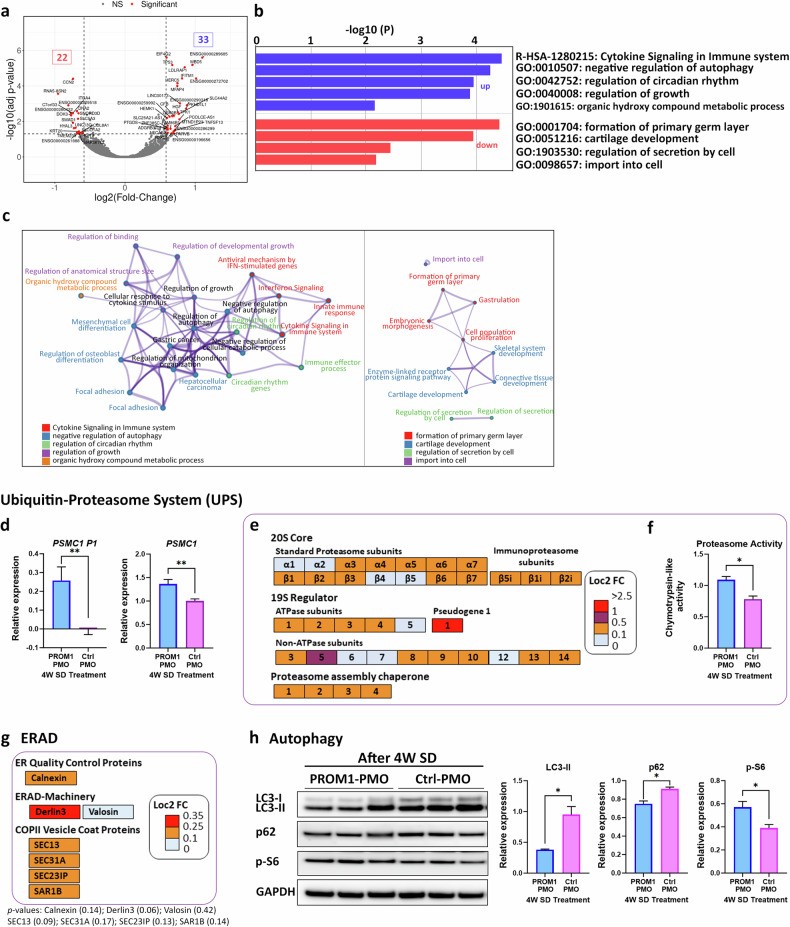
*PROM1* Exon 4 skipping results in upregulation of proteasomal components and downregulation of autophagy. **a** Differentiated expressed genes presented as a volcano plot with the −log10 *p-*value plotted against the log2 FC (PROM1-PMO ROs compared to Ctrl-PMO ROs). Fold-change cut-off was established to 0.585, corresponding to a fold-change of >1.5 and adjusted *p*-value < 0.05. **b** Bar graph of GO terms enriched across upregulated (blue) and downregulated (red) input genes due to exon 4 skipping. Overexpressed genes were mapped to infection process and negative regulation of autophagy, whereas downregulated genes were mapped to developmental processes such as formation of the primary germ layer. **c** Network of enriched terms derived from the upregulated (left diagram) and downregulated (right diagram) genes coloured by cluster ID. Nodes that share the same cluster ID are typically close to each other. Terms with a similarity score >0.3 are linked by an edge (the thickness of the edge represents the similarity score). The network is visualized using Cytoscape. **d** qRT-PCR analysis of *PSMC1* gene and pseudogene expression. Data are shown as mean ± SEM (*n* = 3). Statistical significance was assessed by unpaired Student *t* test. **p < 0.01. **e** PROM1-PMO *t*reatment induced overexpression of several proteasome subunits. The log2FC values are shown on the side of coloured legend, for example orange colour indicates Log2FC > 0.1 and <0.5. **f** Increased Proteasome Trypsin-like activity in PROM1-PMO ROs compared to control confirming RNA-Seq results. Data are shown as mean ± SEM (*n* = 3). Statistical significance was assessed by unpaired Student *t* test. *p < 0.05. **g** PROM1-PMO treatment induced overexpression of several ERAD-related components such as quality control proteins, machinery-related or COPII vesicle coat proteins. The log2FC values are shown on the side of coloured legend, for example orange colour indicates Log2FC > 0.1 and <0.25. *p*-values associated with the Log2FC values are displayed underneath the figure. **h** Representative western blot and quantification analysis of key autophagic components showing downregulation of LC3-II and p62, and upregulation of p-S6 expression in PROM1-PMO treated ROs compared to Ctrl-PMO treated ROs. GAPDH was used as a loading control. Data are shown as mean ± SEM (*n* = 3). Statistical significance was analysed using unpaired Student *t* test. *p < 0.05. ERAD endoplasmic-reticulum-associated degradation system, PSMC1 Proteasome 26S subunit ATPase 1, PSMC1P1 Proteasome 26S subunit ATPase 1 pseudogene 1, p-S6 phospho-S6 Ribosomal protein.

To gain insights into the consequences of exon 4 skipping in the ROs, bulk RNA-Seq analysis was performed in both control and PROM1-PMO treated ROs. A total of 53 genes were significantly differentially expressed (DE) between PROM1-PMO and Ctrl-PMO treated ROs (fold-change of >1.5 and adjusted *p*-value < 0.05, Supplementary Table S1), of which 20 and 33 genes were significantly down- or up-regulated, respectively (Fig. 6a).

Metascape was used to analyse upregulated and downregulated genes separately, identifying five enriched ontology clusters for the upregulated genes, related to “cytokine signalling in immune system” and “negative regulation of autophagy”. For downregulated genes, four different enriched pathways were identified, highlighting the “formation of primary germ layer” and “regulation of secretion by cell” (Fig. 6b). These enriched ontology clusters were also apparent in the enrichment network created by mapping overexpressed and downregulated genes to KEGG pathways (Fig. 6c).

Interestingly, bulk RNA-Seq data presented an outlier (with a log2FoldChange = 2.75) named *PSMC1P1*, Proteasome 26S subunit ATPase 1 pseudogene 1. To confirm the upregulation of *PSMC1P1*, qRT-PCR analysis was performed in both groups of ROs. *PSMC1P1* (pseudogene) and *PSMC1* (gene) have a highly similar coding sequence but differ in their 3’ untranslated region (3’ UTR). Specific primers targeting the identical coding sequence and the unique 3’ UTR were used in the qRT-PCR showing very low expression of the pseudogene, but much higher *PSMC1* expression in the PROM1-PMO treated group (Fig. 6d). *PSMC1* gene encodes one of the subunits (26S ATPase 1) of the proteasome complex, hence RNA-Seq data related to ubiquitin-proteasome system (UPS) were studied in more detail revealing a global overexpression of proteasomal subunits, including the 20S (α and β) and the 19S- subunits, as well as the proteasome assembly chaperones (Fig. 6e). These results were corroborated by proteasome activity assays, which demonstrated higher chymotrypsin-like protease activity in the PROM1-PMO treated ROs (Fig. 6f).

The UPS contributes to the degradation of proteins together with the Endoplasmic-Reticulum-Associated Degradation (ERAD) system. Notably, there was an overexpression of several key components involved in this system such as derlin3, calnexin or COPII vesicle coat proteins (Fig. 6g). As UPS and autophagy are the two key pathways in proteostasis, the autophagy regulation was further studied via western blot. PROM1-PMO treatment significantly decreased p62 and LC3-II levels in the ROs and increased phospho-S6 Ribosomal protein (p-S6) expression, leading to activation of mammalian target of rapamycin complex 1 (mTORC1) (Fig. 6h). This increased expression matches the *TP53* upregulation, which regulates the mTOR complex. These findings collectively suggest a downregulation of autophagy in the PROM1-PMO treated ROs, consistent with the identification of the enriched cluster “negative regulation of autophagy” in the RNA-Seq data.

### Identification of Prominin-1 binding partners using the yeast two-hybrid (Y2H) system

**Fig. 7:**
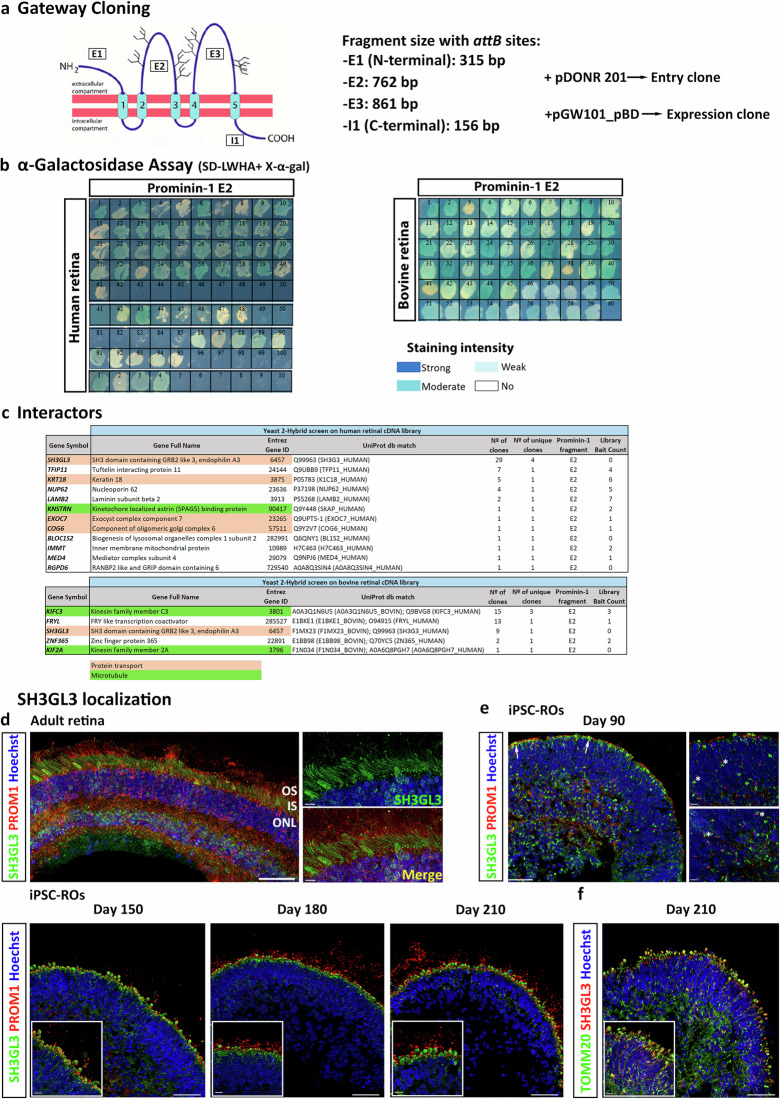
Prominin-1 interactors found in Y2H assay. **a** Schematic representation of Prominin-1 structure and the subdivision into four fragments (E1, E2, E3 and I1) for the Gateway cloning and subsequent screen. Fragment E1: amino acid 2–106; E2: amino acid 180–433; E3: amino acid 504–790; I1: amino acid 814–865. **b** α-Galactosidase Assay, where transformed yeast cells containing both hybrid plasmids (BD and AD recombinant plasmids) were plated in SD-Leu/-Trp/-His/-Ade (SD-LWHA) media with X-α-gal substrate. *MEL1* reporter gene turns X-α-gal substrate into blue coloured end product. **c** Potential Prominin-1 interactors found in the Y2H screen of human and bovine retinal cDNA libraries. Two prominent protein modules can be distinguished: a module composed by proteins linked to protein transport (in orange), and a module of microtubule-associated proteins (in green). Number of clones = number of colonies growing after library mating, sequence verified; Number of unique clones = number of single clones based on sequence**;** Library Bait Count = number of times previously identified in a Y2H screen of this library with an unrelated bait protein. The higher the bait count the less likely that it is specific. **d** Immunofluorescence analysis of SH3GL3 (green) in combination with Prominin-1 (PROM1, red) in adult retina and **e** in hiPSC-ROs during differentiation. ROs were stained at days 90, 150, 180 and 210 of differentiation. Higher magnification images revealed the close association of SH3GL3 with PROM1 at day 90 (white asterisks) and its specific localization in the ISs at later stages. **f** Colocalization of SH3GL3 and TOMM20 in the IS in ROs at day 210 of differentiation. **d**–**f** Cell nuclei were stained with Hoechst. Scale bars: 50 μm; 10 μm (magnification).

To understand the physiological role of Prominin-1 and identify novel binding partners, a *GAL4*-based interaction trap screen in yeast (Y2H system) was used. Using Gateway Cloning strategy, four different plasmids, which have in common the binding domain of *GAL4*, were fused with a different fragment of Prominin-1: Extracellular 1 (E1; which contains the alternatively spliced exon 4), 2 (E2), and 3 (E3) and intracellular 1 (I1) (Fig. 7a). Preliminary experiments showed that Prominin-1 sub-fragments were expressed in yeast without auto-activation, proving that they were good candidates for the Y2H screen. Thus, these four constructs were used in Y2H screening against human and bovine retinal cDNA libraries, respectively. No colonies were obtained for the E1, E3 or I1 fragments neither in the human or bovine retinal libraries.

Using the E2 fragment, a total of 195 clones and 59 clones were isolated during screening of human or bovine retina, respectively, and were subjected to the α-galactosidase colorimetric assay (Fig. 7b). Positives clones from this assay were sequenced, uncovering 12 candidate interactors in the human screening and 5 in the bovine library screening, with SH3GL3 protein present in both (Fig. 7c).

Assessment of the resulting human and bovine candidate interactors revealed four proteins that are involved in protein transport, namely SH3GL3, COG6, EXOC7 and KRT18, some of which are vesicle mediated (SH3GL3 and COG6) or related to Golgi transport (COG6 and KRT18). Other interacting proteins are microtubule-associated, such as KNSTRN, or components of microtubule-based kinesin motors (KIFC3 and KIF2A). Another centrosome-associated protein was found, namely BLOC1S2, a component of the BLOC-1 complex which functions in the formation of lysosome-related organelles. One transcription coactivator, FRYL, involved in cell morphogenesis and neuron projection development was also identified.

As SH3GL3 was identified in both screenings and was highly present in the human screening (29/54 sequences analysed), it was further evaluated by immunofluorescence analysis. SH3GL3 localized to the ISs of the adult retina so no colocalization was observed with Prominin-1 (Fig. 7d). SH3GL3 expression was also assessed at different time points of ROs development, showing colocalization with Prominin-1 in the periphery of the ROs at days 90 and 150 in very close proximity to the ONL at day 90 (Fig. 7e, asterisks). At the latest time points of development (day 180 and 210), SH3GL3 distribution was restricted to the ISs, colocalizing with TOMM20 (Fig. 7f). Further work is needed to validate the interactions of Prominin-1 interacting proteins at the functional level.

## Discussion

Microexons are very short exons, ranging from 3 to 27 nucleotides, and are evolutionarily conserved among vertebrates. Human retina contains the highest number of tissue-enriched microexons, predominantly found in genes involved in cilia biogenesis and vesicle transport, as well as those known to be associated with retinal diseases. Of note, *PROM1* was identified as one of the ciliary genes containing microexons [2]. In a previous study we reported *PROM1* exon 4 inclusion and exon 25 skipping occurring from the 12^th^ week of human retinal development [25]. However, the impact of this specific splicing pattern on Prominin-1 function in the retina has not been addressed. In this study we demonstrate that exon 4 inclusion and exon 25 skipping are neural retina specific, as RPE and other ocular tissues such as lens and cornea, demonstrate lower splicing of these two specific exons during eye development. These splicing events were fully corroborated in the pluripotent stem cell derived ROs. Importantly, exon 4 skipping in ROs at a developmental stage corresponding to onset of inclusion in neural retina in vivo, led to a significant reduction in Prominin-1 expression, defects in cilia formation, shortening of photoreceptor OSs and a significant reduction of cone photoreceptors in the putative ONL. Together these data demonstrate an important role for tissue and stage specific exon 4 inclusion in *PROM1* in photoreceptor development, with cones showing enhanced sensitivity compared to rods.

*PROM1* exon 4 is 27 bp and encodes for nine amino acids (PETVILGLK) of mostly nonpolar, hydrophobic nature localized in the extracellular N-terminal domain. Five of these nine amino acids, namely proline, glutamate, two leucines and lysine (**PE**TVI**L**G**LK**) are conserved across six different vertebrates’ species at positions 93, 94, 98, 100 and 101 respectively [39]. These nine amino acids encoded by exon 4 of *PROM1* form part of an amphipathic helix that could modulate the membrane-binding properties of Prominin-1. In agreement with this notion, it has been postulated that exon 4 residues may function as a spacer element that allows the interaction of Prominin-1 with gangliosides [40]. This interaction may determine the phospholipid composition in the inner leaflet of the plasma membrane, which can affect the shape of highly curved membranes, such as photoreceptor OSs disks.

*PROM1* mutations are associated with a range of inherited retinal diseases including autosomal recessive retinitis pigmentosa with or without macular degeneration and autosomal recessive or dominant cone rod dystrophy [15]. Of the 149 pathogenic mutations linked with retinal disease identified to date, ~23.7% are splicing mutations (Human Gene Mutation Database), which can result in pseudoexon activation [16] or skipping of complete exons, leading to retinitis pigmentosa, retinal macular dystrophy or autosomal recessive cone-rod dystrophy among others (ClinVar 604365). A splicing variant (c.303 + 1G > A) was commonly found in the Spanish population, affecting the canonical donor site of exon 4 and leading to exon 4 skipping in patients with late-onset mild maculopathy [26]. Exon 4 is present in 98% of the Prominin-1 isoforms in the neural retina [14] and thus it has been speculated that it could play an important role in photoreceptor function and maintenance. However, retinal tissue from patients with skipped exon 4 is not available, hence we used pluripotent stem cell derived ROs and a specific PMO that targets the splice acceptor site between intron 3 and exon 4, producing exon 4 skipping, to address the role of this microexon in photoreceptor development.

Our RT-PCR results showed that ES efficiency increased along the incubation period as well as with the number of doses of PROM1-PMO, leading to 57% of *PROM1* exon 4 skipping after one month of incubation and four PMO doses. In agreement, qRT-PCR analysis demonstrated that *PROM1* transcripts with exon 4 were reduced by 55% after the treatment. In silico modelling revealed that Prominin-1 isoform missing exon 4 lacks an internal segment of nine amino acids near the N-terminal, which leads to missing a small helical part in the structure. As the nine amino acids are in frame, we speculated that the isoform would be stable but surprisingly, western blot analysis showed 40% reduction in Prominin-1 expression upon treatment with PROM1-PMO. One explanation is that exon 4 skipping could produce transcripts which are degraded by the nonsense-mediated mRNA decay (NMD) pathway, affecting Prominin-1 expression. However, this was discarded as the transcript levels of *PROM1* mRNA assessed between exons 8 and 12, 20 and 22, and 23 and 25 were not affected after the treatment (data not shown). These results agree with previously published data showing that retinal microexons are unlikely to disrupt open reading frames and affect gene expression through NMD pathway or protein folding [2]. Considering these data, an alternative mechanism may be the cause for reduced expression of Prominin-1.

Notably, Prominin-1 localization was also affected upon the PMO treatment. Prominin-1 is usually localized at the OSs of photoreceptors. In rods, it is concentrated at the base of the OSs, whereas in cones it has a wide distribution throughout the OS lamellar membrane [41]. Our immunofluorescence analysis in ROs at day 180 of differentiation showed that both Prominin-1 antibodies, one recognizing N-terminal domain and a second one recognizing the 1^st^ extracellular loop (E2) colocalized at the OSs; however, this pattern changed after the PROM1-PMO treatment which resulted in 55% exon 4 skipping and 40% reduction in Prominin-1 expression. Four PROM1-PMO doses produced the most drastic effect with Prominin-1 not being transported to the OSs, but instead showing a characteristic circular pattern around the ISs. This suggests that exon 4 skipping and/or reduced Prominin-1 expression is causing the mis-localization of Prominin-1. These results corroborate published data showing lower expression of Prominin-1 with human missense R373C mutation in the retinas of transgenic mice as well as mis-localization of the R373C mutant and wild type Prominin-1 to the myoid region of photoreceptors [18]. As PROM1-PMO treatment also caused a mis-localization of Prominin-1 in the ROs, the idea that it could be retained in the myoid region was explored. Nonetheless, immunofluorescence analysis showed that Prominin-1 did not colocalize with neither myosin VIIa, BiP or GM130, proteins which are located in the photoreceptor myoid region (data not shown). Colocalization was not observed either with TOMM20, a mitochondrial protein, localized in the ellipsoid region.

In addition to decreased Prominin-1 expression, exon 4 skipping resulted in detectable photoreceptor cilium defects such as shortened OSs, reduced ciliation and altered trafficking of cone opsin (opsin R/G). Shortened OSs have already been previously reported in two *Prom1*-knockout mice [42, 43], however as ciliation was reduced, impaired cilia traffic was anticipated. Our data show a significant reduction in the expression of the pan cone marker (*ARR3*) and abnormal accumulation of cone photoreceptors on the basal side of the organoids. Of note, the few cones that were situated in the putative outer nuclear layer displayed an abnormal bulbous OS structure upon the PROM1-PMO treatment, indicating a higher cone sensitivity to *PROM1* exon 4 skipping and/or reduced Prominin-1 expression, in accordance with the reported macular phenotypes [17–20] or cone-rod dystrophies [15, 16, 44, 45] associated with *PROM1* mutations. *Prom1*-knockout mice [43], zebrafish [46] and frog [47] display similar features akin to those obtained in our study including shortened OSs, abolishment of the OS/IS, mis-localization of cone M-opsin, presence of dysmorphic cones, reduction in cone photoreceptors, and retinal degeneration, hence the decrease in Prominin-1 expression could be the underlying factor behind all these observations. To fully dissect whether these effects are specifically due to exon 4 skipping or reduced Prominin-1 expression, further experiments using PMOs that target all isoforms should be performed.

To assess the impact of *PROM1* exon 4 skipping and/or reduced Prominin-1 expression in the ROs we performed bulk RNA-Seq analysis, which revealed an upregulation of several signalling pathways involved in immune response (cytokine and interferon signalling related to primary immunodeficiency response as well as complement activation), cell survival and cell death (p53 signalling pathway, negative regulation of autophagy), metabolism (glycogen metabolism) and protein metabolism (proteostasis). Cellular proteostasis is maintained by two proteolytic pathways: ubiquitin-proteasome system (UPS) and autophagy-lysosome pathway (ALS) [48]. Our RNA-Seq data revealed overexpression of several proteasomal subunits, specifically proteasome 26S subunit ATPase 1, encoded by *PSMC1*, with a fold change >2.5. The induction of proteasomal genes was reflected in an increased chymotrypsin-like activity of 20S proteasome. It is not possible to directly correlate the impact of this subtle change in proteasome activity with the decreased levels of Prominin-1, as the proteasome regulates lot of cellular processes which can contribute to proteins expression [48]. However, previous studies have shown that Prominin-1 undergoes increased ubiquitination when there are mutations in the first ganglioside-binding domain, suggesting a high endocytic turnover [40]. As exon 4 is located very close to this ganglioside-binding domain, we could anticipate that exon 4 skipping would have the same effect in Prominin-1 expression.

Regarding the autophagy pathway, our western blot results demonstrated decreased levels of p62 and LC3-II, and increased levels of p-S6 in PROM1-PMO ROs. Phosphorylation of ribosomal protein S6 at Ser235/236 is a marker of mTORC1 activation [22], so increased levels of p-S6 confirm the *TP53* upregulation found in RNA-Seq data, as one of the downstream targets of p53 is mTORC1. Together, these observations suggest a downregulation of autophagy in in response to exon 4 skipping and/or Prominin-1 downregulation. Published evidence demonstrates that Prominin-1 regulates autophagy flux in the RPE cells via upstream suppression of mTOR signalling and through interactions with p62 and histone deacetylase 6 (HDAC6) [22]. The UPS and autophagy mutually influence each other’s activity via the shared cargo adaptor p62, which levels were decreased after PROM1-PMO treatment. p62 is known to be a negative regulator of the HDAC6 deacetylase activity. A decrease in p62 levels activates HDAC6, decreasing the pool of acetylated tubulin and the polymerization of microtubules, which, in turn, reduce OSs length and alter microtubule-mediated protein transport [49]. These results agree with the shortened OSs observed in PROM1-PMO treated ROs. Furthermore, it is known that Prominin-1 interacts physically with HDAC6, which can negatively influence the dynamics of ciliary length [50]. In accordance, our Y2H screen identified several potential Prominin-1 interacting proteins associated with the centrosome and microtubules, which align with its ciliary localization and a potential role in the photoreceptors’ OSs [40, 50]. In addition, several proteins involved in protein transport were also identified.

The most prominent interactor was endophilin-A3 (endoA3), encoded by *SH3GL3*, which was found in both human and bovine retina Y2H screenings. The potential Prominin-1-endoA3 interaction was further confirmed by immunofluorescence analysis in the ROs, showing colocalization at the periphery of the ROs at days 90 and 150 of differentiation. However, when the ISs and OSs were fully defined in later stages of development as well as adult retina, endoA3 was localized to the ISs and Prominin-1 to the OSs. Our hypothesis is that endoA3 uptakes Prominin-1 from the cell surface during photoreceptors’ development and once they mature, the interaction ceases, releasing Prominin-1 to the OSs, where it may play a role in OSs disk morphogenesis as highlighted by studies in *Prom1*-knockout mice [18, 42]. Supporting this hypothesis, we found that the tumour marker CD166 is a clathrin-independent endocytosis cargo that is internalized in an endoA3-dependent manner [51, 52]. As Prominin-1 and CD166 have shown colocalization at membrane level in colon carcinoma cells [53], it may be that Prominin-1 undergoes transport in a similar manner. Nonetheless, further studies are needed to study the physiological relevance of such interaction.

In summary, the work described herein provides an accessible model for investigating the role of AS of *PROM1* during photoreceptor development but more importantly assessing the impact of splicing associated mutations on photoreceptor development. This model has far reaching impact for the study of other genes containing microexons and involved in inherited retinal disease.

## Supplementary information

Supplementary information

Table S1_1

Table S1_2

Table S1_3

Table S1_4

Table S1_5

Table S1_6

## Data Availability

All the Bulk RNA Seq data have been deposited to GEO under the following accession number: GSE262977.
