# Supplementary material for: Deciphering the impact of PROM1 alternative splicing on human photoreceptor development and maturation
Source: Cell Death Dis. 2024 Oct 1;15(10):721. doi: 10.1038/s41419-024-07105-7 (PMC11445533; doi:10.1038/s41419-024-07105-7)
Supplement: Supplementary file 1 — Supplementary information [file 41419_2024_7105_MOESM1_ESM.docx]

**Supplementary Information**

**Deciphering the Impact of *PROM1* Alternative Splicing on Human Photoreceptor Development and Maturation**

Marina Moya-Molina^1,2^, Birthe Dorgau^1^, Emily Flood^1^, Stef J. F. Letteboer^3^, Esben Lorentzen^4^, Jonathan Coxhead^1^, Graham Smith^1^, Ronald Roepman^3^, Sushma Nagaraja Grellscheid^5,6^, Lyle Armstrong^1^ and Majlinda Lako^1#^

1. Biosciences Institute, Newcastle University, UK
2. Newcells Biotech, UK
3. Department of Human Genetics, Research Institute for Medical Innovation, Radboud University Medical Center, Nijmegen, The Netherlands
4. Department of Molecular Biology and Genetics, Aarhus University, Aarhus C, Denmark
5. Department of Biosciences, Durham University, UK
6. Department of Informatics, University of Bergen, Norway

# to whom correspondence should be addressed:

Majlinda Lako

Biosciences Institute

Newcastle University

International Centre for Life

Newcastle NE1 3BZ

United Kingdom

Email: [majlinda.lako@ncl.ac.uk](mailto:majlinda.lako@ncl.ac.uk)

**
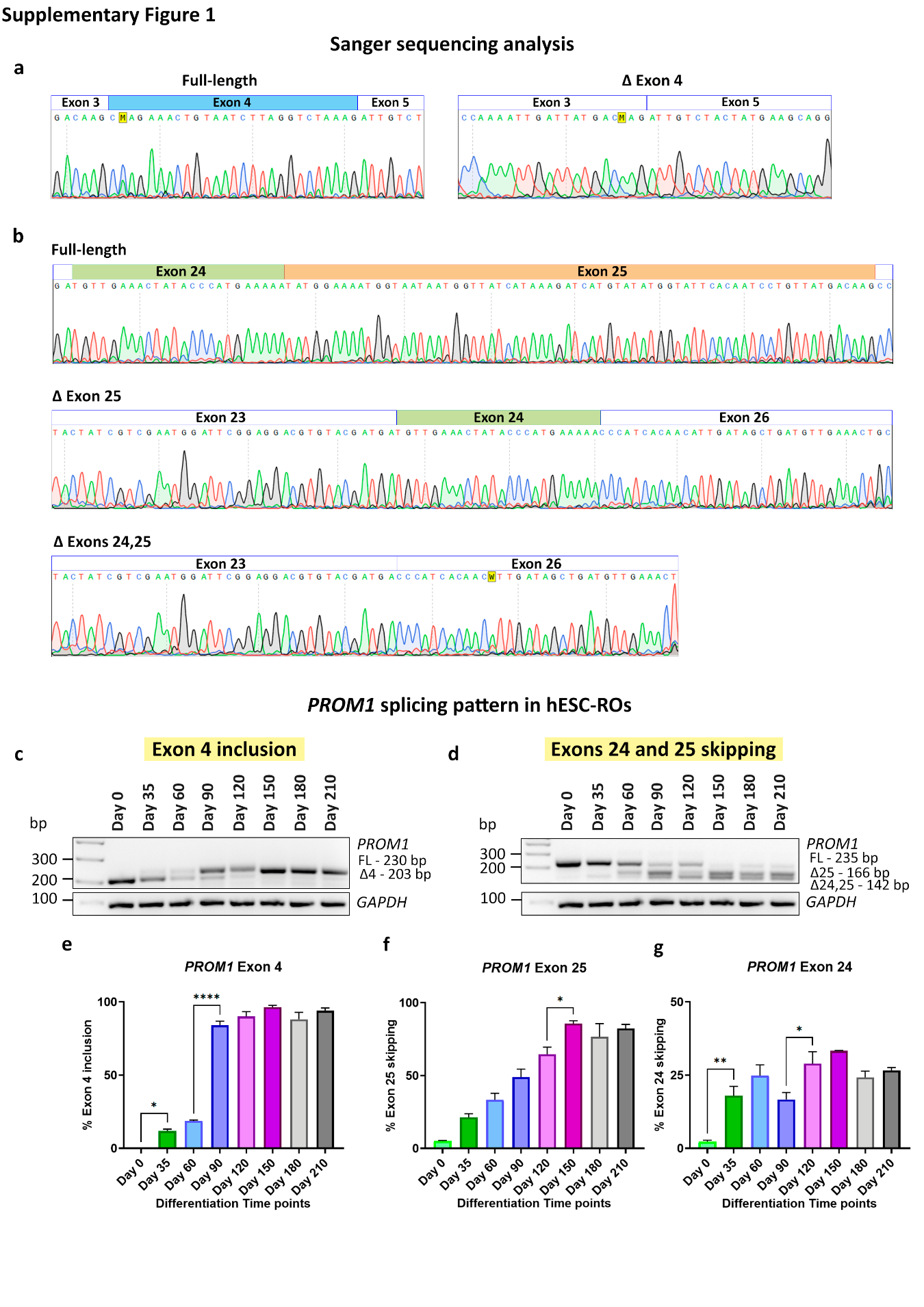
Fig. S1. *PROM1* alternative splicing in hESC-ROs*.*** (**a**) Sanger sequencing analysis of the PCR products obtained using the primers that amplify the region between exons 3-5: full-length product or product missing exon 4. (**b**) Sanger sequencing analysis of the PCR products obtained using the primers that amplify the region between exons 23-26: full-length product, product missing exon 25 or product missing both exons 24 and 25. (**c-g**) RT-PCR analysis of *PROM1* splicing during hESC-ROs differentiation: (**c**) Exon 4 *PROM1* splicing pattern; (**d**) Exon 25 *PROM1* splicing pattern; (**e-g**) Quantification of exon 4 inclusion, exon 25 skipping and exon 24 skipping. Data are shown as mean ± SEM (*n* = 3). Statistical significance was assessed using one way-ANOVA with Sídák’s post hoc test. **p* < 0.05, ***p* < 0.01, *****p* < 0.0001. Abbreviations: FL, Full-length product; 4, Exon 4 skipped; 25, Exon 25 skipped;24,25, Exons 24,25 skipped; PCW, Post-conception week.


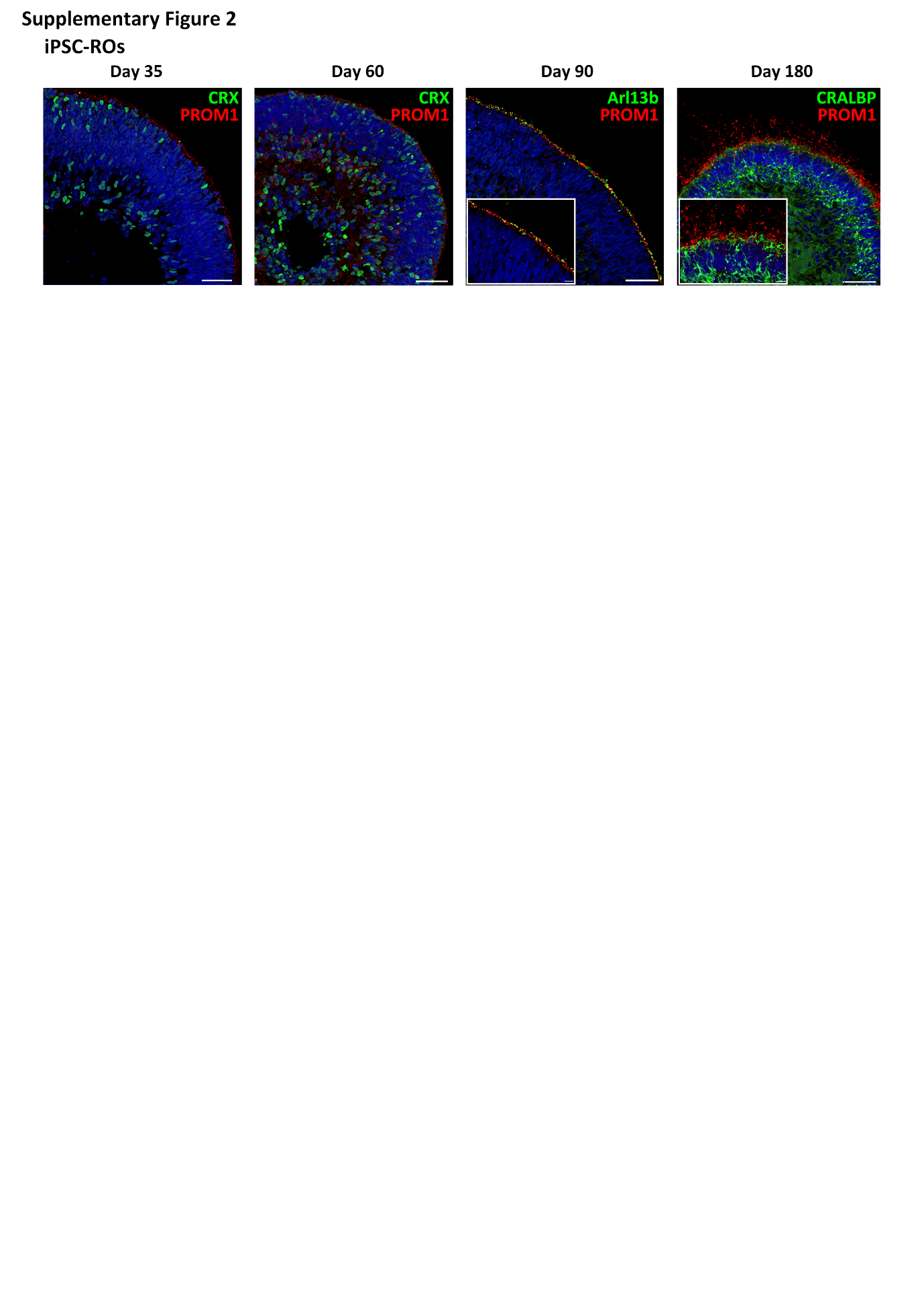


**Fig. S2. Prominin-1 expression in iPSC-ROs at different stages of differentiation.** Immunofluorescence analysis of Prominin-1 during ROs differentiation. At days 35 and 60, Prominin-1 (PROM1, red) antibody was used in combination with CRX (green). At days 90 and 180, PROM1 antibody (red) was used in combination with Arl13b (green) and CRALBP (green), respectively. Higher magnification images revealed the colocalization of Prominin-1 with Arl13b, whereas no colocalization was observed with CRALBP. Cell nuclei were stained with Hoechst. Scale bars: 50 μm; 10 μm (magnification). These are representative images taken from at least 5 ROs/biological triplicate.

**
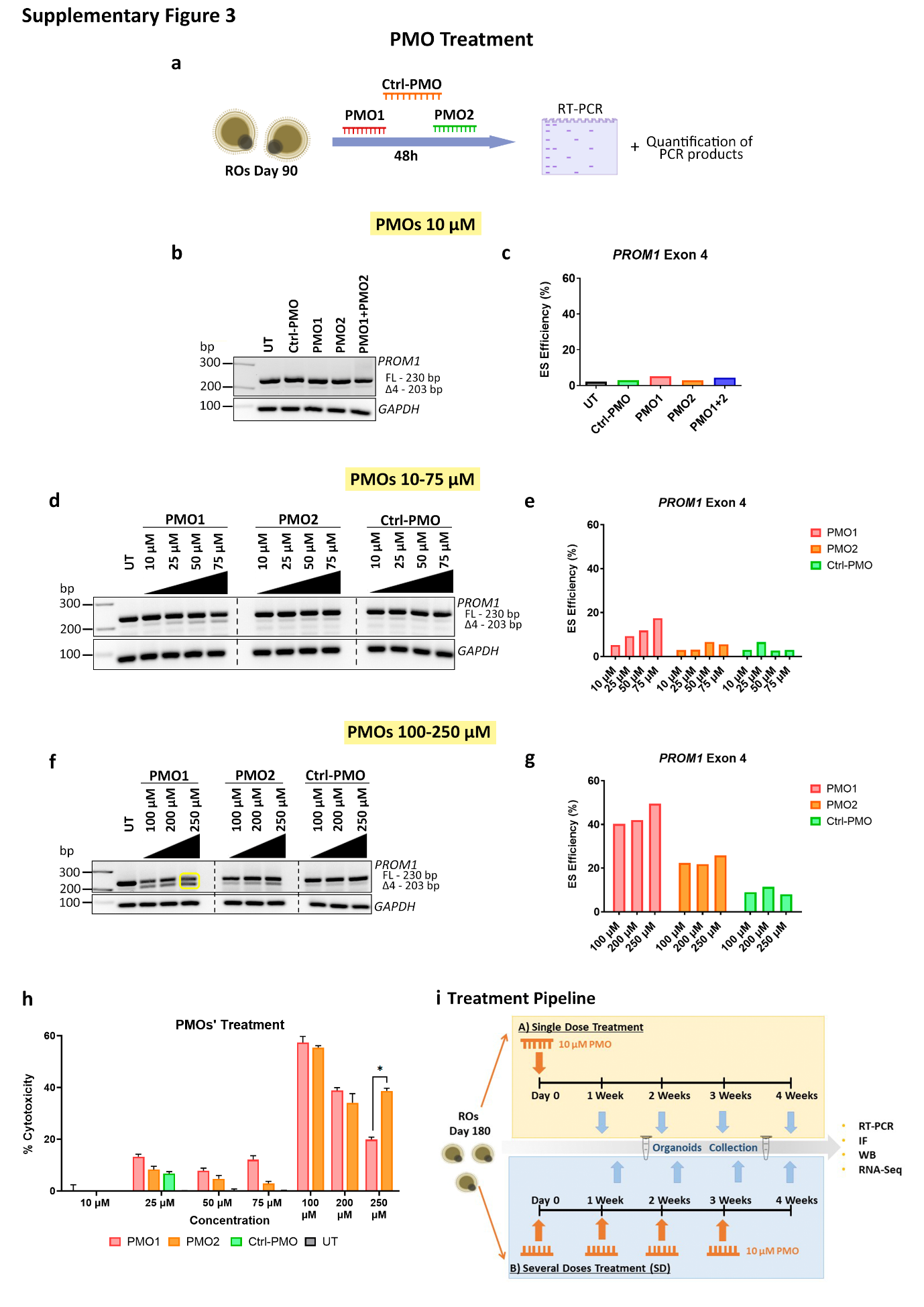
Fig. S3. Screening of different PMO oligos candidates.** (**a**) Schematic representation of the PMO treatment plan. (**b, c**) *PROM1* Exon 4 pattern of ROs after the treatment with 10 μM of PMO1, PMO2 and Ctrl-PMO. (**d-g**) RT-PCR analysis of increasing doses of PMO1, PMO2 and Ctrl-PMO: (**d, e**) 10-75 μM and (**f, g**) 100-250 μM. Densitometric analysis of the gels are shown on the graphs on the right. No statistical analysis was done, just one replicate per treatment. (**h**) LDH assay after the PMO treatment. Data are shown as mean ± SEM (*n* = 3). Statistical significance was analysed by one way-ANOVA with Sídák’s post hoc test. **p* < 0.05. (**i**) Schematic diagram of the treatment pipeline. Abbreviations: PMO, Phosphorodiamidate Morpholino Oligos; Ctrl-PMO, Standard Control oligo; UT, Untreated organoids; FL, Full-length product; 4, Exon 4 skipped.


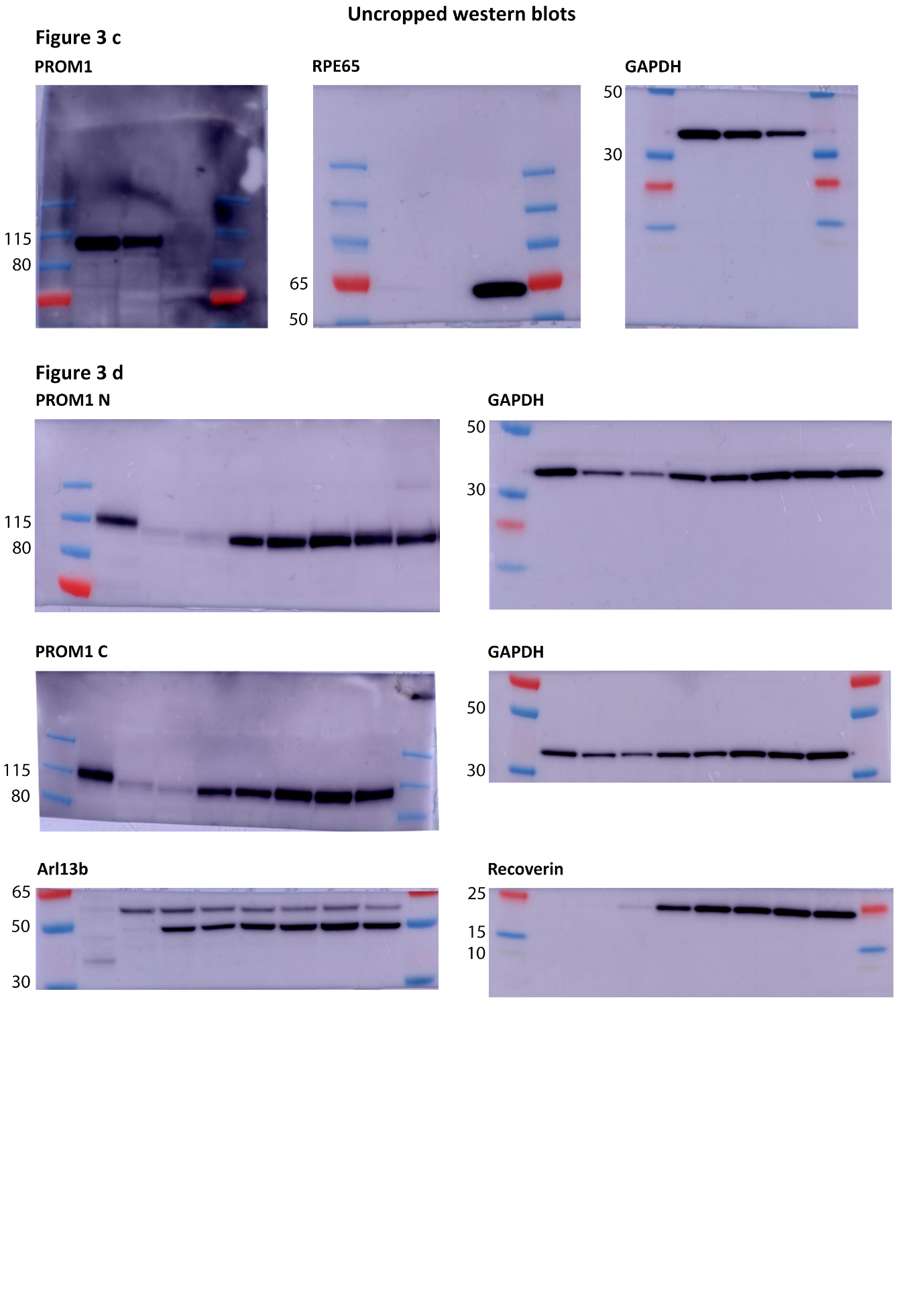


**Fig. S4. Uncropped western blots corresponding to Fig. 3c and 3d.** Please note that some membranes had to be cut to enable multiple antibodies staining.


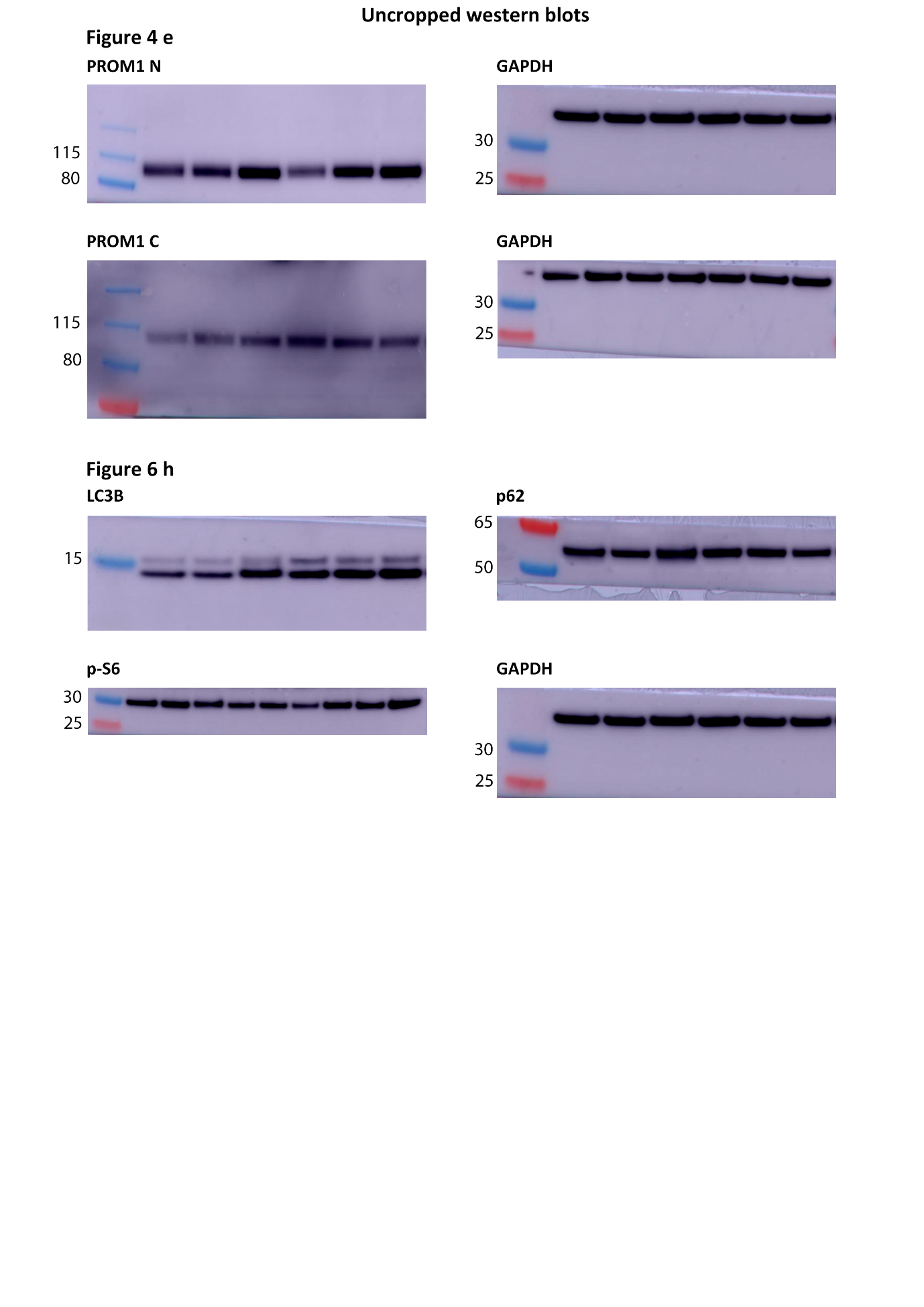


**Fig. S5. Uncropped western blots regarding Fig. 4e and Fig. 6h.** Please note that some membranes had to be cut to enable multiple antibodies staining.

**Table S1:** A summary of reagents and RNA-Seq data shown in different spreadsheets.
